# Chemical Genetics of AGC-kinases Reveals Shared Targets of Ypk1, Protein Kinase A and Sch9*[Fn FN2]

**DOI:** 10.1074/mcp.RA120.001955

**Published:** 2020-02-26

**Authors:** Michael Plank, Mariya Perepelkina, Markus Müller, Stefania Vaga, Xiaoming Zou, Clélia Bourgoint, Marina Berti, Jacques Saarbach, Steven Haesendonckx, Nicolas Winssinger, Ruedi Aebersold, Robbie Loewith

**Affiliations:** ‡ Department of Molecular Biology, University of Geneva, CH-1211, Geneva, Switzerland; § National Centre of Competence in Research - Chemical Biology, University of Geneva, CH-1211, Geneva, Switzerland; ¶ Swiss Institute of Bioinformatics, CH-1015 Lausanne, Switzerland; ‖ Department of Biology, Institute of Molecular Systems Biology, ETH Zürich, CH-8093 Zürich, Switzerland; ** Department of Organic Chemistry, University of Geneva, CH-1211, Geneva, Switzerland; ‡‡ Faculty of Science, University of Zurich, CH-8006, Zurich, Switzerland

**Keywords:** Enzyme inhibition, label-free quantification, phosphoproteome, protein kinases, protein motifs, yeast, AGC-kinases, cell growth, chemical genetics, trehalase

## Abstract

Protein phosphorylation cascades play a central role in the regulation of cell growth and protein kinases PKA, Sch9 and Ypk1 take center stage in regulating this process in *S. cerevisiae*. To understand how these kinases co-ordinately regulate cellular functions we compared the phospho-proteome of exponentially growing cells without and with acute chemical inhibition of PKA, Sch9 and Ypk1. Sites hypo-phosphorylated upon PKA and Sch9 inhibition were preferentially located in RRxS/T-motifs suggesting that many are directly phosphorylated by these enzymes. Interestingly, when inhibiting Ypk1 we not only detected several hypo-phosphorylated sites in the previously reported RxRxxS/T-, but also in an RRxS/T-motif. Validation experiments revealed that neutral trehalase Nth1, a known PKA target, is additionally phosphorylated and activated downstream of Ypk1. Signaling through Ypk1 is therefore more closely related to PKA- and Sch9-signaling than previously appreciated and may perform functions previously only attributed to the latter kinases.

## Abstract (precis)

Analog-sensitive inhibition was used to define phospho-sites regulated by Sch9, PKA and Ypk1. A considerable number of sites affected by Ypk1 inhibition fell into RRxS/T-contexts and overlapped with PKA sites. Neutral trehalase is activated through phosphorylation by both PKA and Ypk1. Signaling through Ypk1 is therefore more closely related to PKA than previously appreciated.

## Graphical Abstract

**Figure:**
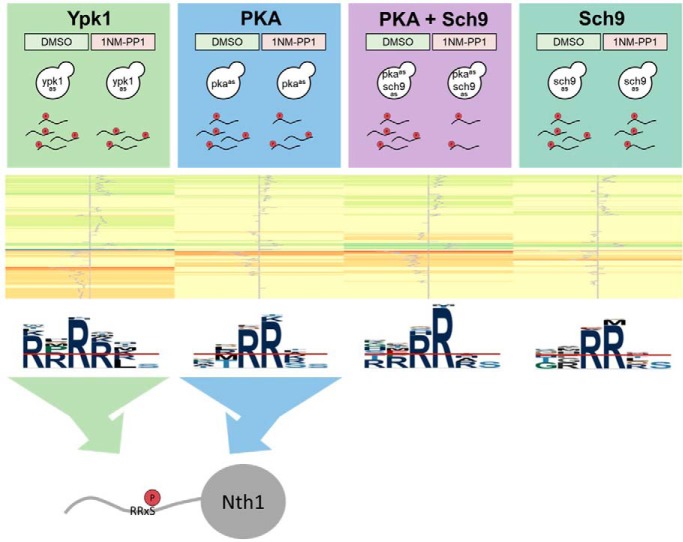

**Highlights**

- Acute inhibition of growth-regulatory AGC-kinases Sch9, PKA and Ypk1.
- Phospho-proteomic profiling of 6373 phsopho-sites.
- Ypk1 regulates phospho-sites in RRxS/T-context and functionally overlaps with PKA.
- Ypk1 and PKA activate trehalase activity of Nth1.

Cell growth is dynamic and highly regulated by signaling pathways that are conserved across evolution. To accomplish this regulation, eukaryotes have developed intricate means to assess growth conditions and to rapidly communicate this information to the processes controlling the accumulation of mass, the modification of cellular volume and of membrane surface area. Although many signal transduction pathways are involved in this regulation, those employing AGC-family kinases (named after Protein Kinases A, G and C) are prominent.

The target of rapamycin complexes 1 and 2 (TORC1 and TORC2) are central sensors of environmental conditions and regulators of cell growth (1). Both complexes exert their functions by phosphorylating AGC-kinases as their main targets. In *S. cerevisiae* TORC1 primarily responds to changes in carbon and nitrogen availability and regulates ribosome biogenesis, cell cycle progression and stress responses via the AGC-kinase Sch9, like S6K downstream of mammalian TORC1 (1, 2).

Another AGC-kinase, protein kinase A (PKA)^1^, performs many, if not most of its functions in parallel to Sch9 by regulating an overlapping set of functions and potentially by cross-talk (3–5). Indeed, Sch9 was originally identified by virtue of its ability to suppress growth phenotypes associated with loss of PKA activity (6). Reciprocally, hyper-activation of PKA signaling can suppress phenotypes linked to the loss of Sch9 activity (4).

Tpk1, Tpk2, and Tpk3 are the partially redundant paralogs of the catalytic subunit of PKA. When cells are starved of carbon, cAMP levels are low and, therefore, PKA is kept inactive by its regulatory subunit Bcy1. Glucose addition induces activation of the adenylate cyclase Cyr1/Cdc35 via the small GTPase Ras1/2 and the G protein-coupled receptor Gpr1. The subsequent increase in cAMP levels triggers the dissociation of Bcy1 from the Tpks allowing them to phosphorylate their substrates (7, 8). Additionally, cAMP-independent activation of PKA has been reported (9). In addition to cell growth, Tpk effectors influence many other processes including carbohydrate metabolism, cell cycle progression, sporulation, pseudohyphal development and longevity by controlling the activities of metabolic enzymes, transcription and autophagy factors (10–13).

Similarly to TORC1, TORC2 phosphorylates and activates AGC-kinases, including Ypk1 and its redundant paralog Ypk2, in their hydrophobic motif (14, 15). Deletion of *YPK2* produces no obvious phenotype suggesting that it only plays a minor role in cell growth control (16). Recent work has demonstrated that TORC2 is regulated downstream of membrane tension (17), oxidative stress (18) and carbon cues (19). In turn, Ypk1, which is homologous to the mTORC2 substrate SGK in humans (20, 21), couples TORC2 signals to the regulation of membrane lipid biosynthesis and the regulation of cell surface area.

Despite their central role in the regulation of fundamental cellular processes and intense efforts in understanding the functions controlled by these kinases, a systematic assessment of their targets has been lacking to date. A previous study aimed at systematically defining changes in the phospho-proteome associated with the absence of protein kinases, using kinase deletion strains (22). However, this approach is limited to the study of non-essential kinases and allows cells to adapt to the absence of a kinase.

To overcome these limitations, we employed yeast strains expressing analog-sensitive (23) variants of PKA, Sch9 and Ypk1. Mutation of the gatekeeper residue of these kinases allows binding of the bulky ATP-analog C3–1′-naphthyl-methyl PP1 (1NM-PP1), thus preventing ATP-binding and rendering the enzymes inactive. Using this selective and acute way of kinase inhibition, we explored the phospho-protein targets downstream of each of these major AGC-kinases by means of quantitative mass spectrometry. In these phospho-proteomics data sets we identified both known and potentially new targets of each tested kinase. As expected, we found extensive substrate overlap between PKA and Sch9. Unexpected was our finding that several substrates were shared between PKA and/or Sch9 and Ypk1 and that many sites hypo-phosphorylated upon Ypk1 inhibition resided in an RRxS/T-motif, which has previously been associated with PKA and Sch9, rather than Ypk1.

Among the numerous potentially new kinase-substrate relationships discovered in this study, we chose neutral trehalase Nth1 as a candidate for follow-up experiments. Nth1 has been employed as a model PKA-substrate in multiple previous studies (24, 25). Trehalose is a disaccharide that functions as a stress-protectant and reserve carbohydrate under adverse conditions (26, 27). Upon return to favorable conditions, trehalose is converted into two molecules of glucose by trehalases, including the neutral trehalase Nth1 (25). The regulation of Nth1 by PKA has long been recognized as important for cell survival (24, 28). Here, we site-specifically validated that Ypk1-, as well as PKA-inhibition reduced its phosphorylation of an RRxS-site and showed that this is associated with reduced trehalase activity.

These findings highlight the need for revisiting the Ypk1 consensus motif and prompt further investigation of the relationship of PKA and TORC1-Sch9 signaling on one hand and TORC2-Ypk1 on the other hand.

## EXPERIMENTAL PROCEDURES

### 

#### 

##### Yeast Cultures and Assays

*Saccharomyces cerevisiae* strains used in this study are listed in Table I. Strains were constructed using standard yeast genetic manipulation (29).

**Table I:** S. cerevisiae strains used in this study

Referred to as	Genotype	Name
pka^as^+sch9^as^	TB50a *tpk1-M164G tpk2-M147G tpk3-M165G SCH9::sch9-T492G(pRS304) ypk1::Kan ypk2::HphNT pRS416-YPK1wt*	MPP88
pka^as^	TB50a *tpk1-as tpk2-as tpk3-as SCH9::SCH9wt(pRS304) ypk1::Kan ypk2::HphNT pRS416-YPK1wt*	MPP86
sch9^as^	TB50a *Sch9::sch9-T492G(pRS304) ypk1::Kan ypk2::HphNT pRS416-YPK1-wt(791)*	MPP84
ypk1^as^	TB50a *SCH9::SCH9wt(pRS304) ypk1::Kan ypk2::HphNT pRS416-ypk1-L424G*	MPP91
pka^as^+ypk1^as^	TB50a *tpk1-M164G tpk2-M147G tpk3-M165G SCH9::SCH9wt(pRS304) ypk1::Kan ypk2::HphNT pRS416-ypk1-L424G*	MPP87
wt^as^ NTH1–3xFLAG	TB50a *SCH9::SCH9wt(pRS304) ypk1::Kan ypk2::HphNT pRS416-YPK1wt NTH1–3xFLAG::HIS3*	yMJP012
pka^as^ NTH1–3xFLAG	TB50a *tpk1-M164G tpk2-M147G tpk3-M165G SCH9::SCH9wt(pRS304) ypk1::Kan ypk2::HphNT pRS416-YPK1wt NTH1–3xFLAG::HIS3*	yMJP010
ypk1^as^ NTH1–3xFLAG	TB50a *SCH9::SCH9wt(pRS304) ypk1::Kan ypk2::HphNT pRS416-ypk1-L424G NTH1–3xFLAG::HIS3*	yMJP009
pka^as^+ypk1^as^ NTH1–3xFLAG	TB50a *tpk1-M164G tpk2-M147G tpk3-M165G SCH9::SCH9wt(pRS304) ypk1::Kan ypk2::HphNT pRS416-ypk1-L424G NTH1–3xFLAG::HIS3*	yMJP040
wt^as^ nth1–5A-3xFLAG	TB50a *SCH9::SCH9wt(pRS304) ypk1::Kan ypk2::HphNT pRS416-YPK1wt nth1-S20A,S21A,T58A,S60A,S83A-3xFLAG::HIS3*	yMJP033
nth1Δ	TB50a *SCH9::SCH9wt(pRS304) ypk1::Kan ypk2::HphNT pRS416-YPK1wt nth1*Δ*::NatNT2*	yMJP013

##### Label-free Phospho-proteomics

Three replicates each of DMSO- and 1NM-PP1- (Merck) treated *sch9^as^*, *pka^as^*, *pka^as^*+*sch9^as^* and *ypk1^as^* cultures were prepared for label-free phospho-proteomics as previously described (30). In brief, all cultures were diluted to OD 0.2 and grown to exponential phase (OD 0.7–0.8). Each strain was treated with 500 nm 1NM-PP1 or DMSO for 15 min after which ice cold 100% TCA was added to a final concentration of 6%. The samples were incubated on ice for 30 min. After centrifugation the cells were washed twice with acetone and kept at −70 °C.

Cell pellets were suspended in a buffer consisting of 8 m urea, 50 mm ammonium bicarbonate, and 5 mm EDTA, then lysed by bead beating. For each biological replicate, 3 mg protein were reduced using 5 mm TCEP, alkylated in 10 mm iodoacetamide, and digested overnight with trypsin (1:125 w/w). Reverse phase chromatography was used to purify samples before phospho-peptide enrichment, which was performed with titanium dioxide resin (1.25 mg resin for each sample) (22). Isolated phospho-peptides were analyzed on an LTQ-Obritrap XL mass spectrometer (Thermo Scientific). A 90-min gradient, starting with 3% and ending with 23% acetonitrile, was used for liquid chromatography elution. The 4 most intense ions detected in each MS1 measurement were selected for MS2 fragmentation.

The mass spectrometry proteomics data have been deposited to the ProteomeXchange Consortium via the PRIDE (31) partner repository with the data set identifier PXD015668 and 10.6019/PXD015668.

##### Data Analysis

Mass spectrometry data were processed with the MaxQuant software (v. 1.6.0.16), which uses the Andomeda MS/MS spectrum search engine (32, 33). The following search settings were used: enzyme was Trypsin/P with 2 allowed missed cleavages. For precursor ions the first search tolerance was set to 20 ppm and the main search tolerance to 4.5 ppm and for fragment ions the tolerance was set to 0.5 Th. Phosphorylation of serine, threonine and tyrosine residues, oxidation of methionine, acetylation of protein N termini and deamidation of asparagine and glutamine were used as variable modifications (maximal 5 modifications per peptide). Carbamidomethylation of cysteine was used as fixed modification. The SGD yeast protein database (https://downloads.yeastgenome.org/sequence/S288C_reference/orf_protein/orf_trans_all.fasta.gz; 6713 entries) from 2015–01-13 was used to match the spectra. Finally, a peptide and modification site FDR of 0.01 was applied to filter peptide spectrum matches (PSMs). Contaminants and decoys were subsequently removed from the results.

The MaxQuant result tables (msms.txt for PSMs and positioning information, modificationSpecificPeptides.txt for intensity values; provided as supplemental Table S1 and S2 respectively) were imported into the R statistical environment to perform missing value imputation and intensity normalization. Only peptides containing at least one phosphorylation were retained, together with all possible positions of the phosphorylation and their respective scores. All intensity values were log_2_ transformed (supplemental Table S3).

Supplemental Fig. S1*A* shows the occurrence of missing values across the 3000+ phospho-peptides revealing that certain peptides and certain MS-runs are more prone to missing values. An example of a peptide with missing values can be seen in supplemental Fig. S1*B*, where all Sch9/PKA-1NM-PP1 values of the peptide HSS(ph)PDPYGINDKFFDLEK and one Sch9 and one PKA value are missing. In the sibling peptide (see below) HSS(ph)PDPYGINDK with clearly higher MS1 intensities all values are present (supplemental Fig. S1*C*). This shows that even if all 3 replicates values are missing, one cannot conclude absence of the corresponding peptide, but these missing values are most likely artifacts inherent to MS1 based quantitation. Phospho-peptides with no missing values in 3 replicates have stronger average MS1 signal intensities compared with peptides with missing values (supplemental Fig. S1*D*). If only one value out of 3 replicate values is missing, the 2 remaining positive replicate values can be used to estimate or impute the missing values (see below). However, if 2 or 3 values are missing, imputation becomes more error prone and can easily lead to false positives, when searching for peptides of differential abundance. Therefore, we only retained peptides with maximally one missing value per 3 replicates. 67.3% of all phospho-peptides fall in this category (supplemental Fig. S1*E*).

We imputed a missing log_2_-transformed intensity value *I_P,S,i_* of peptide *P* in replicate *i* of sample *S* by

(1) $$I_{P,S,i}=\alpha_{P,S,i}+𝒩\left(\mu ,\sigma \right)$$

where α*_P,S,i_* is the mean of the 2 non-missing values of peptide *P* in sample *S* and 𝒩(μ, σ) is a Gaussian error term with mean μ and standard deviation σ. In order to estimate this error term, we first need the notion of sibling peptides. Two peptides are called siblings if the sequence of one peptide is a N/C-terminal extension of the sequence of the other one (*i.e.* they differ by a missed cleavage) and if they carry the same modifications. It is unlikely that the sample condition has a direct influence on the trypsin cleavage, and it can be expected that two sibling peptides have similar expression profiles.

The imputation parameters μ and σ are then estimated using the sibling peptides present in the MaxQuant results: for each peptide *P* with a missing value in replicate *i* in sample *S*, we searched for sibling peptides *P'* of *P* with no missing values in the 3 replicates of sample *S.* Then we calculated the difference between the *I_P',S,i_* and α*_P',S,i_* and used all these differences to estimate μ and σ. The result shown in supplemental Fig. S1*F* reveals that the missing values are on average slightly, but significantly lower than the average of the 2 non-missing values (*t* test *p* value of 0.003), a small bias that we took into account in the imputation Eq. 1).

After missing value imputation, the R package limma (34) was used for intensity normalization. First the *normalizeBetweenArrays* R-function was used to equalize the log_2_-intensity distributions in all 30 LC-MS runs. The lmFit function was used to remove eventual batch effects present in the 10 samples. The empirical Bayes (eBayes) method was the applied to compare the 1NM-PP1 treated to the control (DMSO) samples for each strain and to calculate fold-changes and *p* values adjusted for multiple testing (FDR adjustment) (supplemental Table S3). This method performs a correction of the standard deviation of each peptide by the overall empirical standard deviation.

##### Generation of Sequence Logos

For each phospho-peptide that exhibited a change upon treatment with an adjusted *p* value of less than 0.05 the sequence from −5 to +5 amino acids from each of the phospho-sites localized on the peptide was extracted. In case of ambiguity between isoforms with different phospho-localizations, the peptide with the best localization score was chosen. If a phospho-site was represented by several peptides, the sequence surrounding the site was listed only once. This was performed individually for each strain for hyper- and hypo-phosphorylated peptides and the union of both.

Sequence logos were generated with pLogo using all quantified phospho-peptides as a background (35).

Sequence motifs of sites hypo-phosphorylated upon TORC2-inhibition in Rispal *et al.*, 2015 were based on sites in supplemental Table S2 of that study and generated in an analogous manner (36).

##### Gene Ontology Analysis

For GO-analysis the systematic gene names of proteins harboring phospho-sites significantly changing upon kinase inhibition with an adjusted *p* value below 0.05 were used as a foreground and harboring any quantified phospho-site as a background. The analysis was performed using the “GO Term Finder” tool at the Saccharomyces Genome Database at a *p* value of 0.05 with FDR calculation enabled (37).

The network of proteins associated with GO-term “Endocytosis” in the Ypk1-data set was displayed in the STRING-database tool at default parameters (38).

##### Experimental Design and Statistical Rationale

In the label-free phospho-proteomics, targeted proteomics and the trehalase activity assay, the stated number of replicate cultures of analog-sensitive strains or wt-strain (*wt^as^*) were treated with ATP-analog (1NM-PP1) or mock (DMSO). Three replicates per strain were used for shotgun and targeted proteomics and six replicates for the trehalase assay. Independent starter cultures were used for each strain to generate replicates. The number of replicates used is the highest number deemed justified considering cost and the ability to process samples in parallel. No replication was used in Western blotting as the results were verified by Parallel Reaction Monitoring (PRM) as an alternative approach.

A strain which was not expected to be affected by 1NM-PP1 addition (*wt^as^*) was employed in all experiments. For Western blotting, a strain not carrying a FLAG-tag and another in which five serines of Nth1 had been mutated to alanine were used as additional controls.

For label-free phospho-proteomics, after data processing as described above, the empirical Bayes (eBayes) method of the limma R package (34) was then applied to compare the 1NM-PP1 treated to the mock-treated samples for each strain and to calculate fold-changes and *p* values adjusted for multiple testing (FDR adjustment). Using this approach an observed false positive rate of 0.4% was calculated based on the results from the *wt^as^* strain.

For targeted proteomics and the trehalase activity assay, 1NM-PP1-treated samples were compared with mock-treated samples using unpaired, two-sided Student's *t*-tests for each strain.

When analyzing sets of data points, rather than individual data points (*e.g.* phospho-sites), a trade-off exists between the quality with which each point is measured and the number of points representing each set. To achieve meaningful set-sizes we did not apply further localization score thresholds when analyzing ensembles of phospho-sites (*e.g.* for generating Venn-diagrams or sequence-logos), however, we ensured that sites are based on phospho-isoforms with the highest localization score. As stated in the RESULTS-section, when performing analyses on the level of individual phospho-sites we required the presence of sequence motifs as additional criteria.

##### Parallel Reaction Monitoring

Samples for targeted proteomics by PRM were prepared from 250 μg of protein in a similar manner as for label-free phospho-proteomics, with the exception that a mixture of magnetic TiO_2_, ZrO_2_ and Ti(IV)-IMAC beads (15 μl each; ReSyn Biosciences) were used for phospho-peptide enrichment, essentially following the manufacturer's instructions.

Data acquisition was performed on an Orbitrap Fusion Tribrid mass spectrometer coupled to an nLC1000 uHPLC without use of internal standard peptides (Tier 3). Peptides were separated on a 25 cm EASY-spray column (Thermo Scientific) with a 30 min gradient from 3 to 25% acetonitrile, 0.1% formic acid *versus* 0.1% formic acid in water. MS-scans were acquired at 60 000 resolution, with an ion target of 4 × 10^5^ and maximum injection time of 118 ms. Targeted MS/MS employing higher energy collisional dissociation on 9 selected precursors representing Nth1 phospho-peptides and targeted single ion monitoring on two precursors were each acquired at a resolution of 60,000 with an ion target of 4 × 10^5^ and maximum injection time of 200 ms.

For data analysis in Skyline 4.2.0.19072 we employed spectral libraries from previously generated data-dependent acquisition and filtered for seven b- or y-ions from ion 3 to last ion (39).

The extracted total areas were normalized to the total ion current area of the corresponding sample and an intensity-weighted mean was calculated for cases in which phospho-sites were represented by several precursors. The significance of changes between DMSO- and 1NM-PP1 treated samples was evaluated using a two-sided Student's *t* test.

PRM data are accessible at ProteomeXchange via identifier PXD015760.

##### Spot Assays

Yeast strains were inoculated in YPD (pH 5.6) and incubated at 30 °C and 120 rpm overnight, diluted to OD = 0.1 the next morning and again to 0.0002 in the evening with incubation under the same conditions. Once an OD of about 0.8 was reached cultures were diluted to OD 0.08 and further in a 10x dilution series in the same medium.

For spot assays 3 μl of these cultures were spotted onto YPD agar plates containing 500 nm 1NM-PP1 or an equal volume of DMSO and incubated at 30 °C for 2 days.

##### Determination of Colony Sizes

Yeast strains were inoculated in YPD (pH 5.6) and incubated at 30 °C and 120 rpm overnight, diluted to OD = 0.1, grown to an OD of about 0.8 and diluted to an OD of 0.00008. Of the diluted culture, 100 μl were spread out with glass beads on three YPD-plates per strain and incubated at 30 °C. After 2 days, images were taken and colony sizes quantified using ImageJ (1.52p) (40): After splitting color channels, a threshold was applied to the blue channel to distinguish colonies from background. For each plate the area of colonies was determined using the “Analyze Particles” function set to 25–2000 pixels^2^ and 0.8–1.0 circularity. Overlapping colonies or colonies at the edge of plates were manually excluded. The difference of colony sizes of analog-sensitive strains compared with wt^as^ was evaluated using the Mann-Whitney-Wilcoxon test.

##### Recombinant Protein Purification from S. cerevisiae Cultures

Wt and analog-sensitive (Sch9-T492G, Ypk1-L424G) versions of GST-tagged Sch9 and Ypk1 were expressed in BY4741 from uracil-selectable plasmids employing galactose-inducible promoters: Strains were inoculated in 5 ml SC-Ura + 2% glucose and expanded in 200 ml SC-Ura + 2% raffinose at 30 °C. Protein expression was induced by addition of galactose to 2%. After 3 h, cells were spun down and washed once with cold PBS before snap freezing and storage at - 80 °C.

Pellets were resuspended in 10 ml/g lysis buffer (PBS with 10% glycerol, 5 mm CHAPS, Complete Protease Inhibitor Mixture, 1 mm PMSF) and lysed using a French press (2 rounds, 1100 PSI). Lysates were spun down for 20 min at 20,000 × *g* and 4 °C. The supernatant was incubated with 2 ml glutathione Sepharose 4B beads (GE Healthcare) for 2 h under gentle rotation at 4 °C. Beads were washed once with lysis buffer and an additional three times with lysis buffer containing 350 mm NaCl. Proteins were eluted in lysis buffer containing 50 mm reduced glutathione (pH 8.0), dialyzed against 25 mm HEPES pH 7.5, 150 mm KCl, 2.5 mm CHAPS, 5 mm MgCl_2_, 50% glycerol, adjusted to 60 pmol/μl according to Bradford assay and stored at −80 °C.

##### Recombinant Protein Purification from E. coli Cultures

6xHis-Bmh1 was expressed in BL21 STAR cells from a pET-28b(+) plasmid (a kind gift from the Hahn-lab) (41). Cell were lysed in 50 mm Tris pH 7, 300 mm NaCl, 20 mm Imidazole, 2 mm β-mercapto-ethanol, Complete EDTA-free Protease Inhibitor Mixture, 1 g/l lysozyme by rotation for 20 min at 4 °C and two rounds of sonication for 1.5 min. Debris was removed by centrifugation at 20,000 rpm for 1 h at 4 °C.

Ni-NTA beads (Thermo; 88222) were washed twice in lysis buffer, before incubation with lysate for 1 h at 4 °C. The beads were washed three times with lysis buffer and proteins eluted with lysis buffer containing 500 mm imidazole.

##### In Vitro Kinase Assay

Kinase assays were based on the method by Asensio and Garcia with some modifications (42): Reactions were set up by mixing 10 pmol/μl kinase, 33 pmol/μl Crosstide (43), 100 pmol/μl ATP, 0.02 μCi/μl γ-^32^P-ATP in PBS with 20% glycerol, 0.5% Tween-20, 4 mm MgCl_2_, 10 mm DTT, 2 mg/ml heparin, and Complete Protease Inhibitor Mixture w/o EDTA. After 5, 10, 17, 25, 40, 60 min at room temperature, an aliquot was mixed with 0.5 volumes 0.5 m EDTA with bromphenol blue.

Of the stopped reactions 5 μl were spotted onto P81-paper, which were air-dried, washed five times 10 min with 200 ml 75 mm orthophosphoric acid and once with acetone and dried. An imager screen was exposed to the filter paper for 1 h and the screen read out using a Typhoon FLA 9500 imager (GE Healthcare).

##### Western Blotting

To site-specifically validate changes in Nth1 phosphorylation by Western blotting, yeast cultures were treated with 500 nm 1NM-PP1 or the corresponding volume of DMSO for 15 min and harvested by TCA-precipitation as described for phospho-proteomics sample preparation.

Yeast pellets were lysed by bead beating (5 × 45s at 6500 rpm) in 25 mm Tris buffer containing 6 m urea and 1% SDS. Cleared lysate corresponding to 1.3 mg protein was diluted 1:10 in IP-buffer (50 mm HEPES pH 7.4, 150 mm KCl, 10% glycerol containing protease- (Roche, Basel, Switzerland) and phosphatase- (Thermo Scientific) inhibitors and mixed with 25 μl Epoxy-beads (Thermo Scientific) that had been coupled to anti-FLAG antibody (1.5 μl; Merck) in 0.1 m sodium phosphate buffer containing 1 m ammonium sulfate at 37 °C overnight, and washed four times with IP-buffer.

The lysate was incubated with the beads at 4 °C for 4 h, then washed four times with IP-buffer. Proteins were eluted in 15 μl Laemmli-buffer at 65 °C for 10 min, then in further 15 μl Laemmli-buffer at 95 °C for 5 min.

The eluates from both steps were combined and resolved on a 7.5% SDS-PAGE gel. Proteins were transferred to a nitrocellulose membrane using an iBlot2 system, blocked in 5% BSA in PBS for 1 h. The membrane was incubated with primary antibodies (1:1000 mouse anti-FLAG (Merck) and 1:100 rabbit anti-Nth1-S83p, a kind gift from the Thevelein lab (25)) in 5% BSA in PBS + 0.1% Tween-20 at 4 °C overnight. It was washed four times 5 min in PBS + 0.1% Tween-20, incubated with fluorescently labeled secondary antibodies (LI-COR) in 5% BSA in PBS + 0.1% Tween-20 for 30 min and washed as above. Fluorescent signal was imaged on a LI-COR Odyssey scanner.

##### Trehalase Activity Assay on Permeabilized Yeast Pellets

Measurement of trehalase specific activity was performed as previously described with modifications (44). Six replicates (separate starter cultures) of strains *wt^as^*, *pka^as^* and *ypk1^as^*, as well as one sample of an *nth1*Δ-strain as a control, were inoculated in YPD (pH 5.6) and incubated at 30 °C overnight. Cultures were diluted to an OD of 0.05 and incubated for about 10 h before further dilution to OD 0.001 in YPD. The cultures were grown to an OD of ∼0.7 and, where necessary, cultures were diluted during this time to arrive at a similar OD for all cultures. Subsequently, 100 ml of each culture was centrifuged for 10 min at 2000 × *g*, pellets resuspended in 10 ml YPD and distributed to two 5 ml cultures in 50 ml Erlenmeyer flasks. The cultures were left to recover at 30 °C with shaking at 120 rpm for 25 min before treatment with 500 nm 1NM-PP1 (from a 250 μm stock in DMSO) or the corresponding volume of DMSO. After 15 min of shaking at 30 °C 0.5 ml of each culture were harvested into 0.5 ml of 0.2 m tricine (pH 7.0) with 0.1% Triton-X 100 and 1× phosphatase inhibitor, 200 nm Okadaic acid (Cell Signaling Technology) and 1 EDTA-free protease inhibitor tablet (Roche) per 30 ml, immediately frozen in liquid nitrogen and stored at −80 °C.

Samples were thawed at 30 °C in a thermomixer at 1400 rpm, centrifuged for 1 min at 12,000 × *g* and the pellet washed in 1 ml of cold 0.2 m tricine pH 7.0 (1 min, 12,000 × *g*, 4 °C). Pellets were resuspended in 1 ml of cold 0.2 m tricine (pH 7.0) and transferred into the wells of a deep-well 96-well plate on ice. The plate was spun for 5 min at maximum speed and 4 °C, supernatants removed, and pellets resuspended in 320 μl 62.5 mm tricine pH 7.0, 250 μm CaCl_2_ containing protease- and phosphatase-inhibitors. A well which did not contain a yeast pellet was used as blank. The plate and 0.5 m trehalose were equilibrated to 30 °C for 5 min before 80 μl of 0.5 m trehalose were added to each sample and incubation at 30 °C. After 10, 20, 30, and 40 min a 25 μl aliquot of each sample was transferred into PCR-stripes and heated to 98 °C for at least 3 min.

The samples were cooled to room temperature and cell debris spun down briefly. The amount of glucose in the supernatants was determined using a Glucose-Oxidase/Peroxidase kit (Merck) using 5 μl input in a 150 μl reaction volume.

The amount of glucose generated (= 2× amount of trehalose degraded) was determined against a glucose standard and the blank value subtracted from each sample. The slope of the curve of trehalose degraded *versus* time after trehalose addition with y-intercept of zero was determined as a measure for trehalase activity.

This activity was normalized to the protein level determined by BCA assay (Thermo Scientific) after spinning the remaining samples and bead beating of the resulting pellets in 400 μl 1% SDS, 6 m urea in 25 mm Tris (pH 6.8). The trehalase activity per protein content of the *pka^as^* and *ypk1^as^* strains were each compared with that of the *wt^as^* strain using a two-sided, unpaired Student's *t* test.

##### Trehalase Activity Assays Using In Vitro Phosphorylated Nth1

Recombinant Nth1 purified from *E. coli* (1 μm according to Bradford assay) was mixed with 0.5 μm GST-Ypk1 purified from *S. cerevisiae* or bovine PKA (Promega; V5161) and 300 μm ATP in 25 mm HEPES pH 7.5, 150 mm KCl, 2.5 mm CHAPS, 5 mm MgCl_2_, 1 mm DTT. After 10 min at 30 °C, 12 μm 6xHis-Bmh1 purified from *E. coli* and 30 mm trehalose were added. Samples were incubated at 30 °C and aliquots taken after 5, 10, 20, 30, 40 min, which were heated to 95 °C.

The amount of glucose produced was measured as described above using 5 μl of a 1:100 dilution of the samples as input.

## RESULTS

### 

#### 

##### Evaluation of the Chemical Genetics Approach for the Analysis of S. cerevisiae AGC-kinases

To study substrate specificities and potential signal convergence of growth-regulatory AGC-kinases we used quantitative mass spectrometry to determine changes in the yeast phospho-proteome after acute inhibition of Sch9, PKA, or Ypk1. As overlapping functions of PKA and Sch9 have been reported (3, 6), we also performed simultaneous inhibition of PKA+Sch9. Genes encoding *wt* kinases were replaced with alleles encoding the corresponding analog-sensitive enzymes (34). In the *pka^as^* strains all three PKA-paralogs (*TPK1, TPK2, TPK3*) were replaced with analog-sensitive alleles. The *YPK1* paralog *YPK2*, which is considered to play a minor role compared with *YPK1* (16), was deleted in all strains to avoid redundancy. *S. cerevisiae* strains with analog-sensitive Ypk1 (18), Sch9 (45), and PKA (46) have been used successfully in previous studies.

**Fig. 1.**
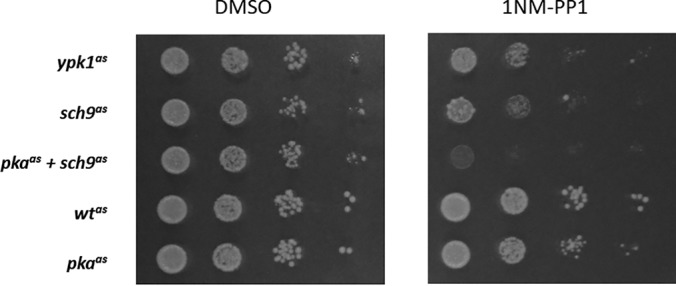
**Inhibition of AGC-kinases inhibits colony formation to varying extents.** Analog-sensitive strains as indicated were spotted on YPD plates containing DMSO or 500 nm 1NM-PP1 in 10x serial dilutions and imaged after 2 days at 30 °C.

We characterized these analog-sensitive strains (denoted as *sch9^as^*, *pka^as^*, *ypk1^as^*, and *pka^as^*+*sch9^as^*) *versus* the isogenic background (referred to as *wt^as^*) by assessing their growth on plates with and without ATP-analog (Fig. 1 and supplemental Fig. S2*A*). Given the central role of all three kinases in growth regulation, we expected visible effects on colony size upon the inhibition of any of them. A slight reduction of colony size was observed for the *sch9^as^* and *pka^as^*+*sch9^as^* strains even on plates without 1NM-PP1, indicating that these strains were mildly hypomorphic. The significance of this effect (*p* < 10^−10^) was verified independently by measuring the sizes of individual colonies when the strains were spread out on YPD-plates (supplemental Fig. S2*B*).

The strongest effect of kinase inhibition on proliferation was seen in case of the *pka^as^*+*sch9^as^* strain, which did not form colonies on plates containing 500 nm 1NM-PP1 (supplemental Fig. S2*A*). Inhibition of Sch9 alone also exhibited a visible reduction in growth. Interestingly, a small number of regular-sized colonies formed under these conditions, indicating that a limited number of cells adapted or became insensitive to Sch9-inhibition. This is consistent with a previous observation in our lab of an *sch9*Δ-strain rapidly generating adapted clones (30). Although less pronounced than for Sch9, the inhibition of Ypk1 also had a strong effect on colony formation on plates.

In contrast, the presence of 1NM-PP1 only mildly compromised colony size in case of the *pka^as^* strain. Therefore, the analog-dependent inhibition of *pka^as^* is either less efficient than for *sch9^as^* and *ypk1^as^* or the absence of PKA activity is compensated by other pathways in our strain background. This second alternative seems unlikely considering the observation that *tpk1*Δ *tpk2*Δ *tpk3*Δ triple-deletion strains are not viable (6, 8, 16). It can be envisioned, however, that there is an increased likelihood of an adaptive response upon prolonged exposure of an analog-sensitive strain to ATP-analog than during creation of a deletion strain. Nevertheless, the comparison of the 1NM-PP1 treated *sch9^as^* and *pka^as^*+*sch9^as^* strain demonstrated that *pka^as^* is sensitive to 1NM-PP1 (Fig. 1 and supplemental Fig. S2*A*) and this finding further suggested that the compensation for PKA function is mainly through Sch9, which is consistent with previous findings (see Broach, 2012 for a review).

##### PKA-, Sch9- and Ypk1-dependent Phospho-proteomes

For phospho-proteome analyses, analog-sensitive strains treated with 1NM-PP1 *versus* DMSO were compared by label-free, quantitative shotgun proteomics.

At a false-discovery rate (FDR) of 0.01, 7575 peptides, 4628 of which were phosphorylated (61%), were identified from phospho-peptide enriched samples from the respective strains. These phospho-peptides corresponded to 6373 phospho-sites (at site-decoy fraction: 0.01), mapping to 1430 proteins.

**Fig. 2.**
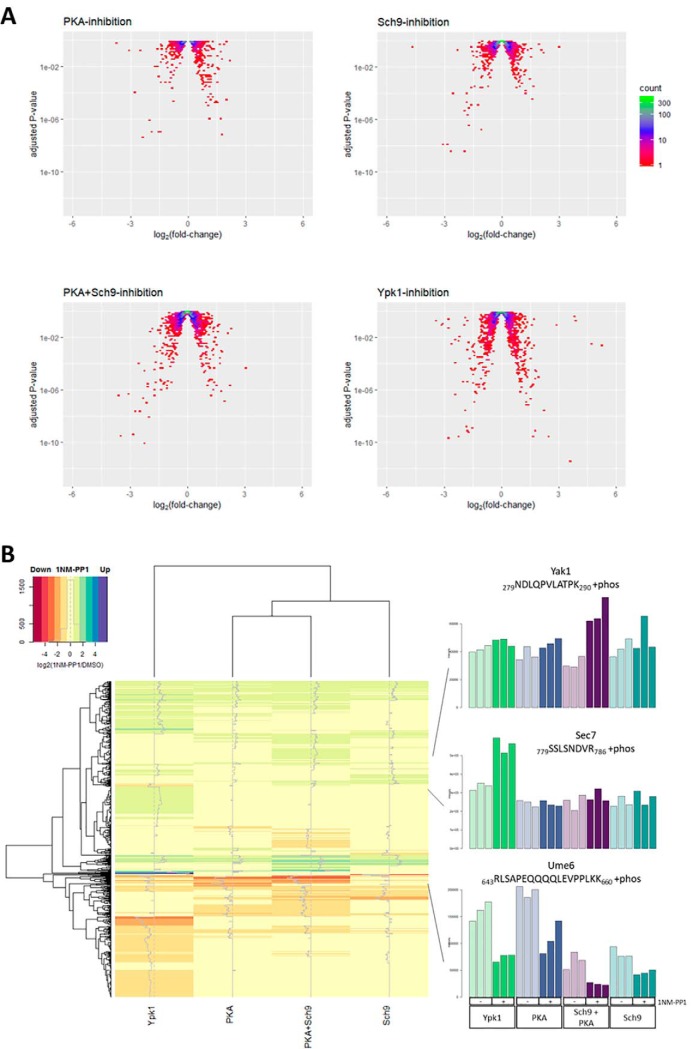
**Phosphorylation changes upon AGC-kinase inhibition.** *A*, Volcano-plots depicting log_2_-fold-changes (mean of three replicates) *versus* adjusted *p* values for intensities of all quantified phospho-peptides in 1NM-PP1 *versus* DMSO treated samples. Density is color-coded according to legend. *B*, Heatmap depicting mean values for three replicates of log_2_-fold changes in phospho-peptide intensities for 1NM-PP1 *versus* DMSO treated samples. Data of phospho-peptides significantly (p_Adj_ < 0.05) hyper- or hypo-phosphorylated upon inhibition of at least one kinase in the PKA+Sch9, PKA, Sch9 or Ypk1 data set are shown. Bargraphs of normalized intensities for three selected phospho-peptides are shown to the right.

Out of the 4628 identified phospho-peptides, 3113 had no more than one missing value in three replicates of any sample and were thus considered for further analysis (supplemental Table S3). An overview of the data as volcano plots is shown in Fig. 2*A*. It is apparent that most phospho-peptides are unaffected by kinase inhibition. At a multiple-testing corrected *p* value cut-off (p_Adj_) of 0.05, 439 phospho-peptides were found to be altered in their abundance upon 1NM-PP1 treatment in the Ypk1-, 128 in the Sch9-, 118 in the PKA- and 293 in the PKA+Sch9 data set (*i.e.* 2.5–9.5% of phospho-peptides were altered in the data sets). For comparison, 12 phospho-peptides were classified as changing when *wt^as^* cells were treated with the ATP-analog, corresponding to a 0.4% false-positive rate.

Principal component analysis of these data revealed that replicate samples clustered, and that separation of samples was mainly explained by genotype rather than by treatment (supplemental Fig. S3). Although the *pka^as^* strain was localized near *wt^as^*, the *ypk1^as^* strain on one hand and *sch9^as^* and *pka^as^*+*sch9^as^* strains on the other formed separate clusters.

With respect to the *sch9^as^* and *pka^as^*+*sch9^as^* strains this result is consistent with the observed reduced colony size of these strains obtained in growth assays in the absence of 1NM-PP1 (Fig. 1 and supplemental Fig. S2*B*). This again suggests that the gatekeeper mutation in Sch9 reduces the catalytic activity of this kinase and renders the strains hypomorphic. In contrast, we did not observe impaired colony formation of the *ypk1^as^* strain. This indicates that the extent of reduced phosphorylation of Ypk1 targets in this hypomorphic strain is not enough to lead to an observable proliferation phenotype.

We argued that the observed hypomorphic phenotypes were because of reduction of kinase activity by the gatekeeper mutations. Indeed, GST-tagged analog sensitive Ypk1 and Sch9 purified from yeast displayed a reduced activity in an *in vitro* kinase assay compared with the respective *wt* versions (supplemental Fig. S2*C*).

A heatmap depicting the intensity fold changes of peptides significantly (p_Adj_ < 0.05) hypo- or hyper-phosphorylated upon kinase inhibition is shown in Fig. 2*B*. This global overview suggests a similar pattern of changes in the PKA+Sch9, Sch9 and PKA data sets, albeit for some phospho-peptides changes are only apparent in the PKA+Sch9 data set. In contrast, the Ypk1 data set contains some phospho-peptides which change in the same direction as in one or more of the other data sets, but the majority appears to change uniquely in this sample.

To further assess the commonalities and differences between the inhibition of these kinases we determined the overlap among significantly (p_Adj_ < 0.05) altered phospho-peptides observed for each strain tested (supplemental Fig. S4*A*).

We first asked whether our data would recapitulate the previously reported interaction between PKA and Sch9 signaling in the form of shared targets (3). Indeed, one third to one half of the phospho-peptides significantly affected (*i.e.* hyper- or hypo-phosphorylated) in one of the data sets was also significant in the other (50 out of 117 and 128 respectively). This may indicate a strong overlap of, or cross-talk between, the signaling pathways. In the former case it can be envisioned that for some shared targets the inhibition of one of the inputs is compensated by the other. An argument in favor of this proposition is the finding that approximately twice as many significant phospho-peptides (293) were identified in the PKA+Sch9 data set compared with the individual PKA and Sch9 data sets. Furthermore, 112 of these were unique to the PKA+Sch9 data set, consistent with the notion that wild type PKA or Sch9 masks the loss of the other for many targets. On the other hand, 80% of the Sch9- and 66% of the PKA-targets were significantly altered also in the PKA+Sch9 data set, arguing that the high number of sites unique to PKA+Sch9 is not merely because of failure to detect true positives repeatedly over data sets.

We next addressed the overlap between PKA/Sch9- and Ypk1-targets, which we expected to be low as TORC2-signaling is less connected to PKA- and Sch9-signaling than the latter two to each other. Consistent with this idea, we found 297 phospho-peptides uniquely affected by Ypk1 inhibition. Surprisingly, 142 phospho-peptides (32%) were shared with at least one other data set, especially with PKA+Sch9 (27%).

The results were qualitatively similar when focusing only on hypo- (supplemental Fig. S4*B*) and hyper-phosphorylated (supplemental Fig. S4*C*) phospho-peptides, in that targets unique to the Ypk1- and PKA+Sch9-data sets constituted the largest groups whereas a strong prevalence of phospho-peptides shared between sets was also apparent.

A full list of gene ontology (GO) terms significantly enriched (*p* < 0.05) among proteins with regulated phospho-sites is given in supplemental Table S4. Among the Biological Process GO terms, “endocytosis.” which was enriched in the Ypk1-data set, is a major downstream process regulated by this kinase (47). Indeed, the majority of the 65 proteins associated with this term form an interconnected network with Ypk1 that contains key proteins from several stages of endocytosis (initiation: Syp1, Ede1; budding: Pan1, Las17, Ent1, Ent2; and coat disassembly: Inp52) (supplemental Fig. S5) (48). It should be noted that many of these proteins likely are not direct Ypk1 targets, but connected further downstream in the Ypk1-Fpk1-Dnf1/Dnf2-Akl1 axis (supplemental Fig. S5) (48). Beyond these fundamental endocytic steps, proteins involved in cargo recruitment (Bul1, Ecm21, Rod1, Aly1, and Aly2) were also found to be affected by Ypk1 inhibition. Interestingly, the nutrient permease regulator Npr1 forms part of this network, even though it previously had been associated with TORC1 rather than TORC2 signaling (49).

“Regulation of transcription involved in meiotic cell cycle” and “Response to abiotic stimulus” were both enriched in the PKA-, whereas no significant terms were found for the Sch9- and PKA+Sch9-data sets.

Similar gene ontologies related to transferase/glucosyltransferase activity were detected for the PKA, Sch9 and PKA+Sch9 data set and the proteins associated with these GO-terms in the three data sets strongly overlapped and included subunits of the trehalose 6-phosphate/phosphatase complex (Tsl1 and Tps3), glycogen phosphorylase Gph1 and glycogen synthases Gsy1 and Gsy2 (supplemental Table S4). The fact that both homologs Gsy1 and Gsy2 were detected based on distinct peptides lends validity to the finding that the regulation of storage carbohydrate metabolism is an important function of PKA and Sch9 signaling (supplemental Table S3). Additionally, the neutral trehalase Nth1 was found to be affected by PKA and Ypk1 inhibition (see below).

In summary, we observed that all the analog-sensitive kinases were affected by 1NM-PP1 addition based on the growth of the corresponding strains and on observed changes in protein phosphorylation. These phosphorylation changes are consistent with previous knowledge, especially with respect to the close connection between PKA- and Sch9-signaling. Additionally, enriched GO-terms reflect previous data, but are represented also by additional proteins that had not been linked to the respective kinases.

##### Sequence Motifs

**Fig. 3.**
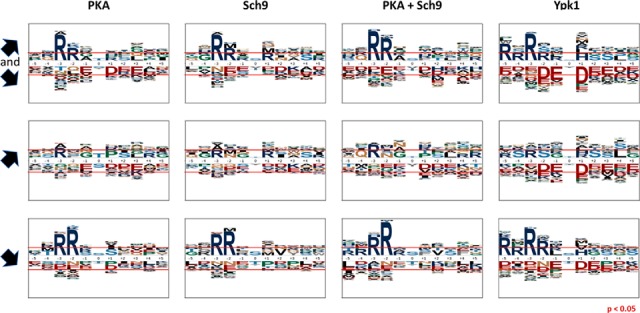
**AGC-kinase target sites are located in basic sequence motifs.** Sequence motifs from -5 to +5 amino acids surrounding serines and threonines significantly (P_adj_ < 0.05) affected by inhibition of PKA, Sch9, PKA+Sch9 and Ypk1 (left to right). Top to bottom rows: Union of hyper- and hypo-phosphorylated, hyper-phosphorylated, hypo-phosphorylated sites. Red horizontal lines indicate over- and underrepresentation at *p* = 0.05.

To determine if AGC-kinases in our experiments preferentially targeted specific amino acid sequences, we generated logos of the sequences surrounding phospho-sites significantly affected by 1NM-PP1 treatment. This was done individually for up- and down-regulated sites and for a combination of both for each of the four data sets (Fig. 3).

A clear enrichment of arginines in the -2 and -3 position of residues hypo-phosphorylated upon the inhibition of any of the kinases was observed. The motifs of the PKA, Sch9 and PKA+Sch9 data sets closely resembled each other in this respect and exhibited stronger enrichment of arginine in the -2 than -3 position. These findings are consistent with the reported RRxS/T consensus motif for PKA and Sch9 and may therefore reflect the presence of direct targets of the studied kinases (50, 51).

For hypo-phosphorylated sites in the Ypk1-data set in contrast, arginine was more strongly enriched in the -3 position and further enrichment of arginines N-terminal of the phosphorylated residue, at -5 and -2, could be observed. The sequence context determined for Ypk1 fitted the reported RxRxxS/T-motif because of the enrichment of arginine in the -5 and -3 position (52), however additionally an unexpectedly high frequency of arginine in the -4 and especially -2 position was apparent in our data. As Ypk1 is a major target of TORC2, we compared this motif to the sequence motif of sites found hypo-phosphorylated upon TORC2-inhibition in a previous study (36). A strong prevalence of arginines in the -5, -3 and -2 positions was also observed in this data set (supplemental Fig. S6), which argues that the sequence specificity of Ypk1 was not altered because of the gatekeeper mutation.

It is noteworthy that a slight enrichment of arginine in the -3 position of sites hyper-phosphorylated upon kinase inhibition was observable. As the change in abundance was opposite to what was expected for direct targets, it is possible that this residue forms part of the recognition sequence of a kinase that is itself repressed by the analog-sensitive kinases studied.

In each case, the PKA+Sch9 motifs constituted a more pronounced version of the PKA and Sch9 motifs, therefore indicating that Sch9- and PKA-signaling converge either through shared targets or cross-talk.

##### Phosphorylation Levels of Known Direct AGC Kinase Targets

To estimate if our approach was suitable to identify direct AGC-kinase targets we asked how many of their previously known target-sites were identified and exhibited significant down-regulation upon kinase inhibition in our data set. Ypk1, Sch9 and PKA target sites derived from the literature are listed in supplemental Tables S5*A*–S5*C*.

**Fig. 4.**
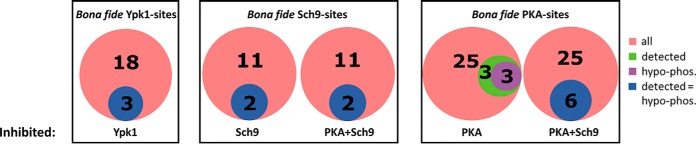
**Detected *bona fide* kinase target sites are largely found hypo-phosphorylated upon kinase inhibition.** Venn diagrams depicting the number of *bona fide* target sites of the kinase indicated (red), subset of these sites detected by phospho-proteomics (green) and subset of these exhibiting significant (p_Adj_ < 0.05) hypo-phosphorylation (purple). Blue circles indicate a complete overlap of the two subsets.

Although only 14% to 19% of *bona fide* kinase target sites were detected (*i.e.* identified and quantified) by mass spectrometry, most of these sites were significantly hypo-phosphorylated upon inhibition of the respective kinase:

Three of the 21 known Ypk1 target sites were detected in this study and all found to be down-regulated upon Ypk1-inhibition (Lag1 S24, Gpd1 S24, Rod1 S138; Fig. 4). Similarly, two of the 13 known Sch9 target sites were detected here and both were down-regulated in the Sch9 and the PKA+Sch9 data sets (Stb3 S254, Maf1 S90; Fig. 4). Finally, six of 31 *bona fide* PKA-sites were detected and three of them were down-regulated in the PKA (Bcy1 S145, Nth1 S60, Nth1 S83) and six in the PKA+Sch9 (Bcy1 S145, Nth1 S60, Nth1 S83, Maf1 S90, Pat1 S456 and Pat1 S457) data set (Fig. 4).

Therefore, although our phospho-proteomics data suffered from the high false negative rate with respect to detection, typically associated with this approach, the quantitative accuracy appeared sufficient to detect biologically meaningful changes.

##### Many Proteins Possess More Than One Regulated Phospho-site

Several of the previously reported AGC-kinase targets (*e.g.* Fpk1, Orm1, Fps1, Stb3, Maf1, and Rim15) harbor more than one regulated phospho-site (supplemental Table S5A to S5C) (53–58). We therefore asked if this feature could also be observed in our study. Site-specific quantification of phosphorylation levels can be complicated by several sites residing on the same phospho-peptide as it may not be clear which site is responsible for an observed change in phospho-peptide abundance. Furthermore, sites located near lysine or arginine may impede tryptic cleavage and distort the quantification of nearby residues. We therefore used the presence of arginine in the -3 position from the phosphorylated residue, which is a frequent feature of target sites of the AGC-kinases under investigation, as an additional criterion for selecting proteins with multiple regulated sites (21, 59).

Notable observations included the detection of two regulated RxxS-sites on the previously reported Sch9-target Stb3 in the Sch9 and PKA+Sch9 data sets (56). We also observed two and three regulated phospho-sites on the protein kinase Ksp1 in the Sch9 and PKA+Sch9 data sets respectively, one of which (S624) was also significant in the Ypk1 data set. Additionally, S884-phosphorylation on this protein was only affected upon Ypk1-inhibition. In the Ypk1 data set, only a few of the RxxS/T-sites on multi-site proteins were found in the strict RxRxxS/T-motif ascribed to Ypk1 (59). Instead, several of the multi-site proteins were shared with other data sets, including Smy2, Gcs1, Igo2, Ksp1, and Avt1 (supplemental Table S3).

Similarly, several of the regulated RxxS/T-sites on multi-site proteins in the PKA data set were found in relaxed motifs, *i.e.* without a basic residue in the -2 position, but with a proline at +1 (T70 and S96 on Smy2; T161 and T170 on Gcs1; S135, S147, and S161 in Tsl1). Interestingly, four regulated phospho-sites were detected on the trehalose synthase subunit Tsl1 in all four data sets.

Finally, three RxxS phospho-sites on Bcy1 were significantly affected in the PKA and PKA+Sch9 data sets. Phospho-peptides containing either S83 or S145 exhibited an ∼45–75% reduced abundance upon kinase inhibition in both data sets, whereas phospho-peptides with T129 showed an up to 2-fold increase in the PKA data set.

As Bcy1 is the common regulatory subunit of all three catalytic PKA-subunits (Tpk1, Tpk2 and Tpk3), it is conceivable that these sites form part of a feedback loop regulating PKA activity. Indeed, S145 has previously been reported as a PKA target as part of its negative feedback mechanism (60). The same study identified S145 as the by far major target of PKA on Bcy1, which is consistent with this site being the only RRxS/T-site in the protein. The other sites determined in our study may therefore be targets of other kinases or weaker PKA-targets.

We did not detect any peptides on phospho-diesterase Pde1, which has been reported as another component of cAMP-signaling that is a target PKA (61). Instead, S534 and S631 on Ras GTPase activating protein Ira2 were significantly altered in their phosphorylation state upon PKA inhibition and may therefore constitute a so far unknown feedback mechanism of PKA.

The reason for the clustering of phosphorylation-sites on AGC-kinase targets is not well understood in most cases, but it may allow generating switch-like responses similarly to other kinase targets (62). In any case, the discovery of multiple sites within certain proteins, which are affected by kinase inhibition suggests that the function of these proteins is regulated by said kinase.

##### Phospho-sites Within Kinase Consensus Motifs

As described above, we observed a strong enrichment of arginine in the -3 position of phospho-peptides significantly hypo-phosphorylated upon inhibition of any of the kinases tested. Indeed, of the 484 identified phospho-sites within a RxxS/T-motif, 117 were significantly (P_adj_ < 0.05) hypo-phosphorylated in at least one of the data sets. Because this is consistent with previously reported kinase consensus motifs (RRxS/T for Sch9 and PKA and RxRxxS/T for Ypk1) (51, 59), we argued that the corresponding sites may constitute potential direct kinase substrates. We asked if these potential direct targets are shared between kinases tested or specific to individual kinases.

**Fig. 5.**
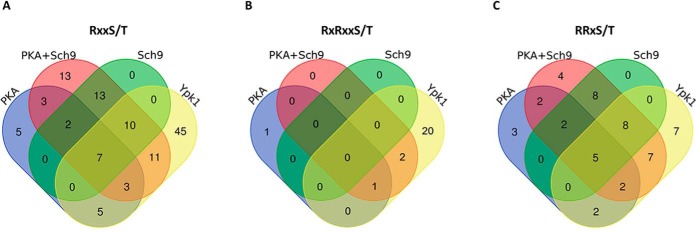
**Overlap of hypo-phosphorylated sites associated with kinase consensus motifs between kinase inhibition data sets.** Venn-diagrams represent phospho-sites significantly (p_Adj_ < 0.05) hypo-phosphorylated upon inhibition of PKA, PKA + Sch9, Sch9 or Ypk1 in RxxS/T- (*A*), RxRxxS/T- (*B*) and RRxS/T- (*C*) motifs.

Like the data on all hypo-phosphorylated sites, the vast majority of RxxS/T-sites in the PKA or Sch9 data set were also part of the PKA+Sch9 set. Not all of these sites overlapped between the PKA and Sch9 samples, indicating that they may be targets of only one of the two kinases or that inhibition of one kinase targeting a site is compensated by the second kinase (Fig. 5*A*).

Although 45 RxxS/T-sites were uniquely affected by Ypk1-inhibition, 36 sites were also hypo-phosphorylated in at least one other data set. As the Ypk1 consensus motif is distinct from the PKA/Sch9 motif, we asked if this overlap mainly comprised sites within RRxS/T- or RxRxxS/T-motifs.

We therefore evaluated the overlap of significantly hypo-phosphorylated RxRxxS/T and RRxS/T sites between data sets (Fig. 5*B* and 5*C*). As expected, the majority of RxRxxS/T-sites (20 of 24) were unique to the Ypk1-set. Consequently, sites with an RxRxxS/T-motif do not explain the overlap of RxxS/T-sites between the Ypk1 and the other three data sets.

On the other hand, 31 RRxS/T-sites were hypo-phosphorylated in the Ypk1 data set, of which 24 overlapped with at least one of the other sets (Fig. 5*C*). Among these 31 sites only four were simultaneously in an RxRxxS/T-motif (*i.e.* RxRRxS). Assuming these sites are indeed direct Ypk1 targets, the previously defined Ypk1 consensus motif of RxRxxS/T may need to be reconsidered.

We next asked if the RRxS/T-sites that are potential Ypk1 targets had any other sequence characteristics close to the phospho-site, but no obvious amino acid enrichments at other positions within five residues from the phospho-site were observed (supplemental Fig. S7).

Following the unanticipated observation that Ypk1 affects the phosphorylation state of sites presumed to be PKA- and/or Sch9-targets based on our results and their sequence context, we next aimed to validate this observation on a selected candidate protein.

##### Nth1 Phosphorylation of S83 is Positively Regulated by PKA and Ypk1

Our phospho-proteomics assay detected a phospho-peptide (_81_RGSEDDTYSSSQGNR_95_ + phos) of neutral trehalase Nth1 with potential phosphorylation at S83 or T87 (or a mix of both), down-regulated with a fold-change (FC) of 0.6 (*p* = 0.007), 0.5 (*p* = 0.009) and 0.3 (*p* = 4 × 10^−5^) on Ypk1, PKA and PKA+Sch9 inhibition respectively. A second phospho-peptide of Nth1 mapping to S60 (_58_TMSVFDNVSPFKK_70_ + phos) exhibited significant down-regulation upon Ypk1 (FC = 0.4; *p* = 0.0004), PKA (FC = 0.5; *p* = 0.01) and PKA+Sch9 (FC = 0.5; *p* = 0.02) inhibition.

Nth1 is a commonly cited model-substrate of PKA and its trehalase activity is increased by PKA-dependent phosphorylation of its N-terminal tail (28, 63, 64).

In contrast, although Ypk1 had previously been shown to regulate metabolic functions, including of enzymes influencing the levels of the osmo-protective metabolite glycerol, the regulation of the major thermo-protective metabolite trehalose has not been reported (65, 66). To validate our phospho-proteomics results we asked whether Ypk1-inhibition would alter the phosphorylation of an RRxS-site (S83) on Nth1, whose phosphorylation had previously been attributed to PKA (25, 64).

**Fig. 6.**
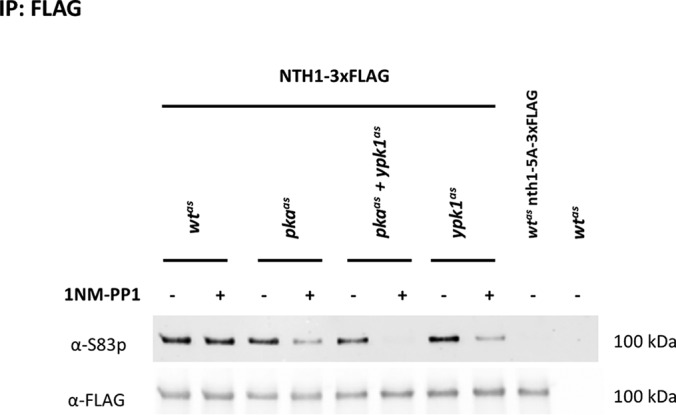
**Western blotting demonstrates dephosphorylation of Nth1 S83 upon inhibition of PKA, PKA+Ypk1 and Ypk1.** Strains *wt^as^*, *pka^as^*, *pka^as^*+*ypk1^as^* and *ypk1^as^* harboring endogenously C-terminally 3xFLAG tagged Nth1, the corresponding isogenic wt strain with 3xFLAG-tagged nth1-S20A,S21A,T58A,S60A,S83A (*wt^as^* nth1–5A-3xFLAG) and untagged isogenic wt strain (*wt^as^*) were treated with DMSO or 500 nm 1NM-PP1 for 15 min. Eluates from immuno-precipitates with an anti-FLAG antibody were probed with antibodies against S83p and against the FLAG-tag.

**Fig. 7.**
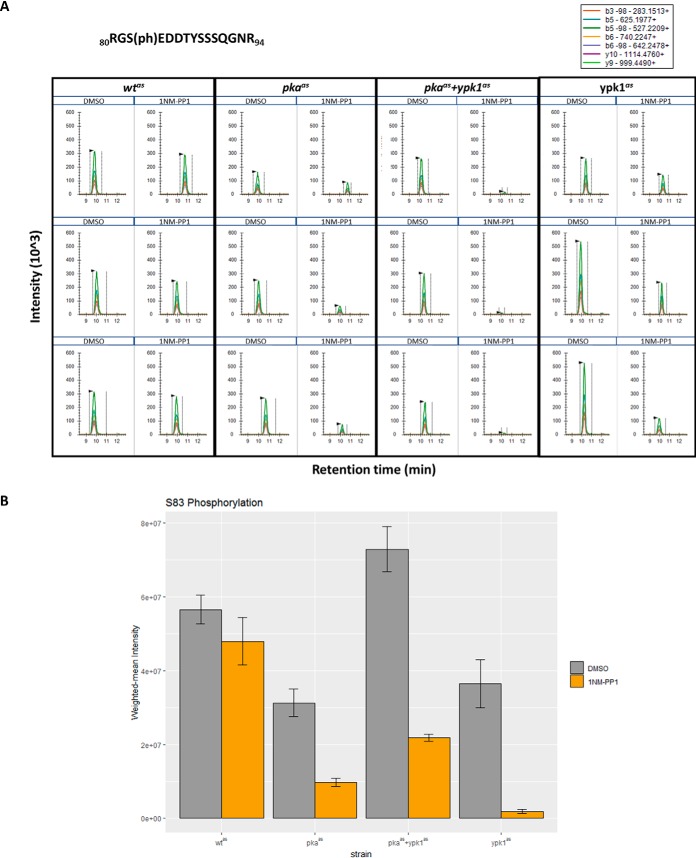
**Parallel reaction monitoring demonstrates dephosphorylation of Nth1 S83 upon inhibition of PKA, PKA+Ypk1 and Ypk1.** *A*, Extracted ion chromatograms of transitions of seven site-determining ions (*i.e.* ions not shared with other potential phospho-isoforms) of _80_RGS(ph)EDDTYSSSQGNR_94_ (precursor 580.2270; z = 3) were extracted from a PRM-experiment on phospho-peptides from strains treated with DMSO or 500 nm 1NM-PP1 for 15 min. Three replicates were analyzed per condition. *B*, Barplot depicting weighted-mean intensities of phospho-peptides corresponding to Nth1 S83p shown in A. Indicated strains were treated with DMSO (gray) or 500 nm 1NM-PP1 (orange) for 15 min. Bars represent averages from three replicates and errorbars represent standard error of the mean. Asterisks indicate significant fold changes at *p* values less than 0.05.

We evaluated the phosphorylation of this site on endogenously 3xFLAG-tagged Nth1 after immuno-affinity purification by Western blotting with an antibody against phosphorylated S83 (S83p) (25). Indeed, the signal was reduced upon 1NM-PP1 treatment of strains harboring either analog-sensitive Ypk1 or PKA (Fig. 6). Importantly, no signal remained in the absence of both enzymatic functions. We also tested the phosphorylation of S83 upon Ypk1, PKA and Ypk1+PKA inhibition in an independent experiment using a targeted proteomics assay. The results closely agreed with the ones obtained by Western blotting and confirmed that S83, and not T87, was the phosphorylated residue (Fig. 7*A* and 7*B*).

These findings validate the effect of Ypk1 on phosphorylation of a site located within an RRxS-motif and therefore not assumed to be a direct Ypk1 target based on its previously reported consensus motif (59).

##### Nth1 is Activated in a PKA- and Ypk1-dependent Manner

It has been previously reported that the PKA-dependent phosphorylation of the N-terminal tail of Nth1 stimulates the enzyme's trehalase activity (24, 25, 28). Based on our results of Ypk1-dependent phosphorylation of Nth1 we asked if Ypk1 would also regulate Nth1 activity.

For this we again compared *wt^as^*, *pka^as^* and *ypk1^as^* strains treated for 15 min with 1NM-PP1 with mock-treated controls and assessed trehalase activity by a method similar to the one described by De Virgilio, 1991 (44).

**Fig. 8.**
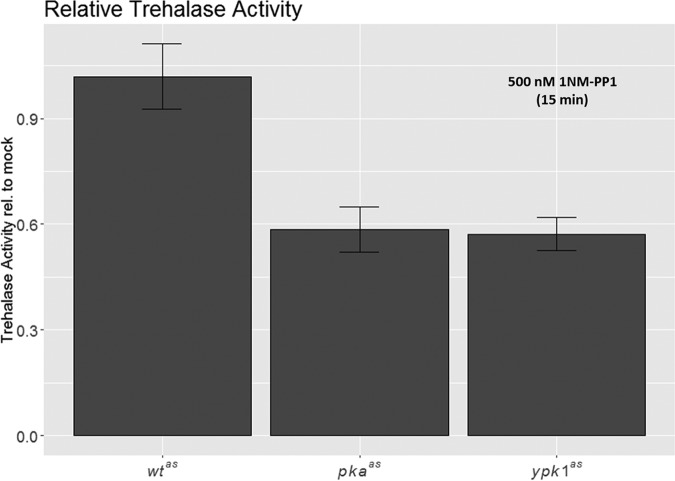
**Inhibition of PKA and Ypk1 leads to reduced Nth1 activity.** Barplot depicting trehalase activity of 1NM-PP1 treated *wt^as^*, *pka^as^* and *ypk^as^* strains relative to activity of corresponding DMSO-treated strains. Strains were in exponential growth phase and treatment was for 15 min. Error-bars depict standard deviation of six replicates. Reduction of relative trehalase acitivity in *pka^as^* and *ypk1^as^* strains is significant with *p* = 6 × 10^−6^ and *p* = 1 × 10^−5^ respectively.

We employed a *nth1*Δ strain as a control in this experiment. For this strain no detectable trehalase activity could be observed (supplemental Fig. S8*A* and S8*B*) indicating that Nth1 is the only trehalase active under these conditions. Note that acidic trehalase is not expected to be active at the neutral pH used for this assay. We observed that trehalase activity was strongly reduced in the *pka^as^*, and somewhat also in the *ypk1^as^* strain, even in the absence of 1NM-PP1, indicating the hypomorphic nature of these strains (supplemental Fig. S8*A* and S8*B*). Importantly, a significant reduction of trehalase activity in both analog-sensitive strains was observed upon 1NM-PP1 addition relative to mock treated controls (*p* = 6 × 10^−6^ and 1 × 10^−5^ respectively; Fig. 8, supplemental Fig. S8*A* and S8*B*). This indicates that, as for PKA, Nth1-activity was positively regulated in a Ypk1-dependent manner.

**Fig. 9.**
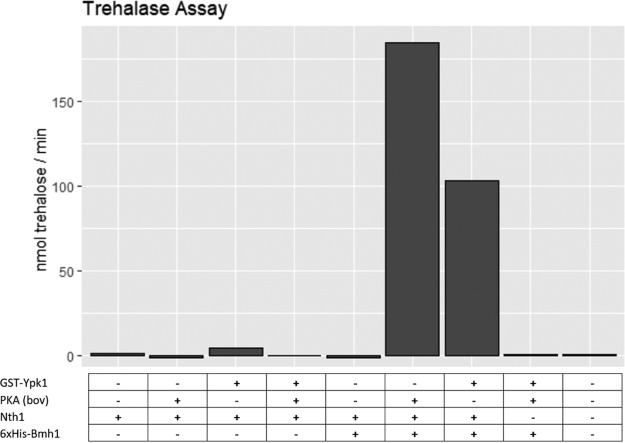
**PKA and GST-Ypk1 each activate Nth1 trehalase activity in presence of 6xHis-Bmh1.** Recombinant Nth1 was incubated with ATP and GST-Ypk1 or PKA as indicated, before addition of trehalose and 6xHis-Bmh1 as indicated. Trehalase activity was determined by measuring the amount of glucose produced over time.

Finally, we tested if Ypk1 can activate Nth1 in a direct manner. To this end, we incubated Nth1 with GST-Ypk1 and ATP before measuring trehalase activity. Indeed, similarly to PKA, GST-Ypk1 strongly induced trehalase activity (Fig. 9). This increase in activity was dependent on the presence of Bmh1, a 14–3-3 protein reported to bind phosphorylated Nth1, as previously reported (63).

It can therefore be envisioned that stresses inactivating Ypk1 contribute to trehalose accumulation. Vice versa, return to favorable growth conditions and re-activation of Ypk1 may contribute to trehalose degradation by Nth1. Lastly, basal Ypk1 activity appears to contribute to basal Nth1 activity in exponentially growing cells.

## DISCUSSION

### 

#### 

##### Previous Studies on AGC-kinase Functions

In this study we investigated the targets of three major growth-regulatory AGC-kinases in *S. cerevisiae*, PKA (Tpk1, Tpk2, Tpk3), Sch9 and Ypk1. For this purpose, we employed analog-sensitive versions of these kinases in combination with phospho-proteomics. A *ypk1*Δ *ypk2*Δ double- and *tpk1*Δ *tpk2*Δ *tpk3*Δ triple-deletion renders cells inviable and deletion of *SCH9* leads to severe phenotypic effects, demonstrating the important roles that these kinases perform (6, 8, 16). A previous large scale phospho-proteomics investigation of strains in which individual kinases had been disrupted included *tpk1*Δ, *tpk2*Δ, *tpk3*Δ, *ypk1*Δ, and *ypk2*Δ strains (22). Of necessity, this approach precluded the study of strains harboring co-deletions of essential paralogs. A further disadvantage of using deletion strains for studying an enzyme is that such strains inevitably adapt in ways that often mask effects on direct targets.

Analog-sensitive strains of AGC-kinases have been described previously, but not to determine the effect of their inhibition on global phosphorylation changes (17, 45, 67). We reasoned that the acute inhibition of kinases via a chemical-genetics strategy in combination with a direct readout of phosphorylation changes should enable us to increase our understanding of signaling downstream of these kinases and in particular identify novel direct targets.

##### Interplay Between Sch9- and PKA-signaling

There is ample genetic evidence for a close link between PKA- and Sch9-dependent signaling (3). In fact, Sch9 was identified as a high copy suppressor of a temperature sensitive mutation in the Ras2-guanine nucleotide exchange factor Cdc25 (6). On the flip-side, Sch9 exhibits synthetic lethality with components of the PKA-pathway, Gpr1, Gpa2, and Ras2 (68–70). Signaling through the PKA-pathway represses stress-protective activities of Yak1 and Msn2/4 and G0-entry via Rim15. The subsequent heat-shock sensitivity of an activating mutation in *GPA2* is suppressed by deletion of *SCH9* (71).

Consistently, we observed a strong overlap of phospho-sites affected by PKA- and Sch9-inhibition. Further, a high number of sites detected only upon simultaneous inhibition of both kinases may comprise further shared sites for which the activity of one kinase masks the inhibition of the other. The consensus sequence detected for both kinases closely resembles their established RRxS/T-motif (51, 72, 73).

These observations pose fundamental questions for future research: Is the considerable overlap between sites affected by PKA and Sch9 inhibition because of cross-talk between the two signaling pathways or because of shared targets of the two kinases? In case of targets unique to one of the kinases, how is this selectivity established, considering the high similarity of the PKA- and Sch9-consensus motifs? The data provided in this study serves as a starting point to answer these questions.

##### Interplay Between Ypk1- and PKA/Sch9-signaling

Although evidence for an overlap between PKA- and TORC1-Sch9-signaling had been reported, TORC2-Ypk1-signaling is considered to be distinct from the former two pathways. Despite shared subunits between the TOR-complexes, TORC1 is believed to mainly respond to nutritional cues whereas TORC2 receives its input primarily from the cell membrane (74) as well as glucose (19).

Several findings in this study tie Ypk1 much more closely to PKA and Sch9 than we previously assumed: whereas many phospho-peptides were affected uniquely upon Ypk1 inhibition, a surprisingly high number overlapped with the other data sets. Second, although the Ypk1 consensus motif derived in this study was consistent with the previously reported RxRxxS/T-motif, a clear enrichment of arginine in the -2 position could be observed. Closer investigation of basophilic sites hypo-phosphorylated upon Ypk1 inhibition revealed two nearly mutually exclusive sets of Ypk1 targets: one set of sites associated with the RxRxxS/T-motif and almost uniquely affected by Ypk1-inhibition and the other of sites associated with the RRxS/T-motif and largely shared with at least one other kinase. These observations suggest that Ypk1 recognizes two related, but distinct motifs. Alternatively, Ypk1 may regulate these sites indirectly through an RRxS/T-specific kinase.

We detected 92 phospho-sites within RRxS/T-motifs out of 1041 such sites in the iPTM-database (75) (and 1241 occurrences of the motif in the yeast proteome). Thus, a sharper picture may emerge if data on these missing sites is acquired. Beyond the incomplete nature of any phospho-proteomics study, it is possible that the choice of trypsin as a protease is a limiting factor as phospho-peptides originating from basic sequence contexts may frequently be too small for reliable identification by MS. Although the use of trypsin may therefore cause blind-spots, it is not expected to bias the observed motifs as they were constructed against a background of all peptides quantified in this study.

##### Kinase Target Motifs

The commonly cited Ypk1 target motif is RxRxxS/T (with an additional preference for a hydrophobic residue in the +1 position) and was defined by the Thorner lab based on nine *bona fide* Ypk1 target sites on five proteins and previously published *in vitro* assays on synthetic peptides (46). This resembles the consensus motif of the presumed human homolog of Ypk1/2, SGK (21). It is interesting to note that, although one of the *in vitro* studies to define the Ypk1 target motif did not test peptides containing the RRxS/T-sequence (51), the second reported a Ypk1 motif similar to our study: whereas arginine in the -3 position was most strongly enriched, a similar prevalence of arginine in -5 and -2 was also observed (51, 72). Parenthetically, for Ypk2 the target sequence even more closely resembled an RRxS/T-motif (72). Collectively, these results suggest that Ypk1 also recognizes RRxS/T-motifs besides RxRxxS/T.

After the initial definition of the Ypk1 consensus motif (52) 12 further sites on six proteins were identified, which all reside in RxRxxS/T-motifs (52, 52, 55, 76). However, the presence of this motif in each case constituted a criterion in the search for new Ypk1 sites, biasing the selection.

It should also be noted that the assays performed in our study targeted Ypk1 during conditions of basal activity rather than in contexts where it is highly activated. It may be speculated that full activation of Ypk1 leads to a change in its substrate preference. This idea is particularly intriguing as distinct phosphorylation steps by Pkh1/2 and TORC2 may lead to partial and full activation of Ypk1 (14, 21).

In contrast to Ypk1, the sequence motif for sites hypo-phosphorylated upon PKA-inhibition closely agrees with the previously reported PKA-consensus motif. Similarly, our Sch9-motif is in agreement with a motif previously defined by peptide-array (51) and with the fact that *bona fide* Sch9 target sites are generally in an RRxS/T-motif (2, 30, 56).

##### Regulation of Trehalose Metabolism by Ypk1

We further investigated the connection between PKA- and Ypk1-signaling by choosing neutral trehalase Nth1 as an example. The phosphorylation of its N-terminal tail (S20, S21, S60 and S83) by PKA has been previously reported (25, 28, 64). It has been suggested that this phosphorylation leads to the activation of its trehalase activity, mainly through the binding of 14-3-3 proteins to phosphorylated S60 and S83 (63, 64). We here showed that Nth1 phosphorylation and activity is also diminished by Ypk1 inhibition *in vivo* and that Nth1 is activated by Ypk1 *in vitro*, therefore establishing a connection between TORC2-signaling and storage carbohydrate metabolism.

Few Ypk1 targets are currently known. Regulation of trehalose metabolism may therefore be a so far overlooked TORC2-dependent stress response. If this regulation is indeed via direct phosphorylation of the N-terminal tail of Nth1 by Ypk1, remains to be determined. The fact that two of the presumed regulatory sites, S20 and S60, reside within an RxRxxS/T-sequence, the classical Ypk1 target motif, argues in favor of this possibility, regardless of if Ypk1 can also phosphorylate RRxS/T-sites.

Based on the finding that trehalose prevents the aggregation of partially unfolded proteins, but also interferes with their re-folding, it has been proposed that rapid activation of trehalase in heat-shock recovery is important to restore protein function (27). We speculate that activation of trehalase by Ypk1 may be primarily of importance under conditions when PKA activity is low, but to date failed to demonstrate this physiological role. Alternatively, activation of Nth1 may be a combined function of phosphorylation by PKA and Ypk1. This proposition is attractive because of the presence of preferred PKA and Ypk1 sites in the Nth1 N-terminal tail, according to their classical consensus motifs.

Further phospho-sites on trehalose-6-P synthase/phosphatase subunits Tsl1, Tps2 and Tps3 were affected by Ypk1 inhibition and may provide further ways for TORC2-signaling to modulate trehalose metabolism.

The TORC2-dependent regulation of endocytosis in contrast is well-established, however mechanistically not well understood (47). Our data provide an extensive list of Ypk1-regulated phospho-proteins involved at several steps of endocytosis. It will be interesting to map the location of these proteins in the TORC2-Ypk1 pathway and to define phenotypic consequences of site-specific mutants of the corresponding phospho-sites.

Finally, we reported phospho-sites affected by the inhibition of AGC-kinases on proteins that had not been previously linked to signaling through these kinases. Based on the close match of sequence motifs derived from these sites and the reported consensus motifs of the kinases it is likely that we detected several direct kinase targets, setting the stage for future focused studies.

## DATA AVAILABILITY

DDA data have been deposited to the ProteomeXchange Consortium via the PRIDEpartner repository with the data set identifier PXD015668 and 10.6019/PXD015668. Spectra were additionally deposited to MS-Viewer and are accessible using key 99yh6tladc.

Supplemental Table S1 is available at https://doi.org/10.6084/m9.figshare.11474685.

PRM data have been deposited at Panorama Public: https://panoramaweb.org/wMKBjd.url and are accessible at ProteomeXchange via identifier PXD015760.

## Supplementary Material

Table_S2

Table_S4_and_S5

Table S3

Supplementary figures

## Footnotes

^1^ The abbreviations used are:

PKA: Protein kinase A
AGC-kinases: Protein kinases of the family comprising PKA, PKG and PKC
TORC1/TORC2: TOR complex 1/2
1NM-PP1: C3–1′-naphthyl-methyl PP1
FDR: false-discovery rate
GO: gene ontology
PSM: peptide spectrum match.
