# Supplementary material for: Chemical Genetics of AGC-kinases Reveals Shared Targets of Ypk1, Protein Kinase A and Sch9
Source: Mol Cell Proteomics. 2020 Feb 26;19(4):655–71. doi: 10.1074/mcp.RA120.001955 (PMC7124472; doi:10.1074/mcp.RA120.001955)
Supplement: Supplementary figures [file 158471_0_supp_465349_q4rvdt.docx]

Chemical genetics of AGC-kinases reveals shared targets of Ypk1, Protein Kinase A and Sch9

**Michael Plank, Mariya Perepelkina, Markus Müller, Stefania Vaga, Xiaoming Zou,** **Clelia Bourgoint, Marina Berti, Jacques Saarbach, Steven Haesendonckx, Nicolas Winssinger, Ruedi Aebersold, Robbie Loewith**

**Supplementary materials included in this file: Fig. S1-S8**

**Supplementary tables included in separate files:
 Table S1 (TabS1_msms.xlsx)
 Table S2 (Tab_S2_modificationSpecificPeptides.xlsx)
 Table S3 (Tab_S3.xlsx)
 Table S4-S5 (Tab_S4toS5.xlsx)**

| A  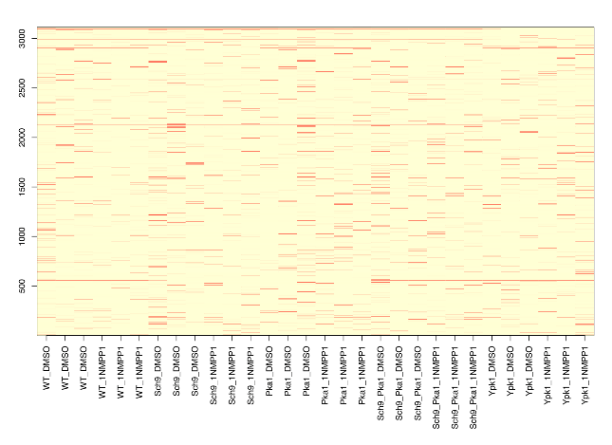 | B 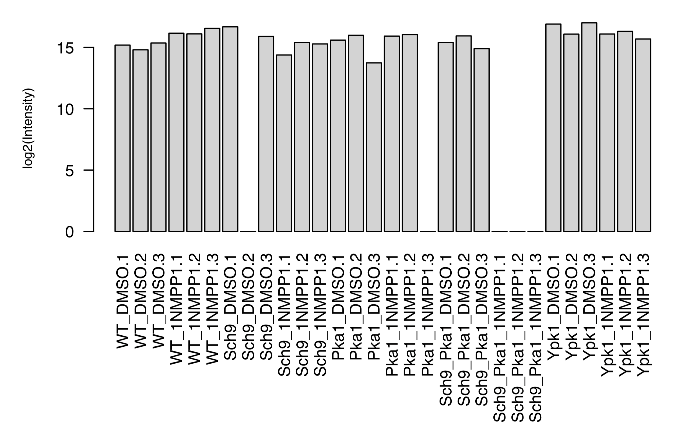 |
| --- | --- |
| C 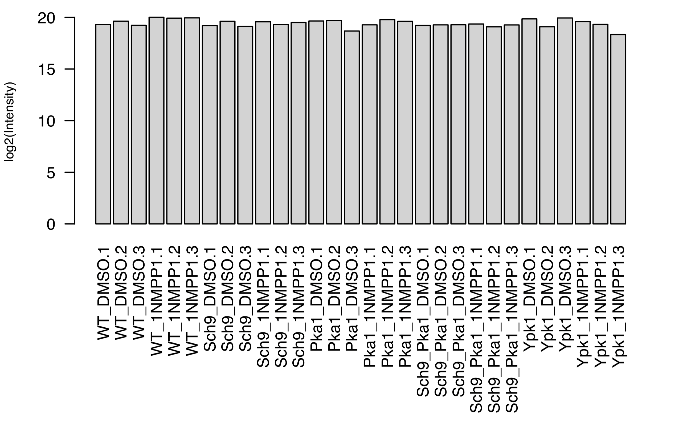 | D  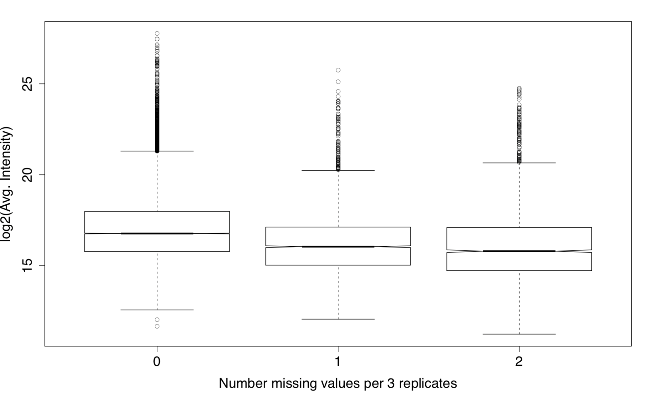 |
| E 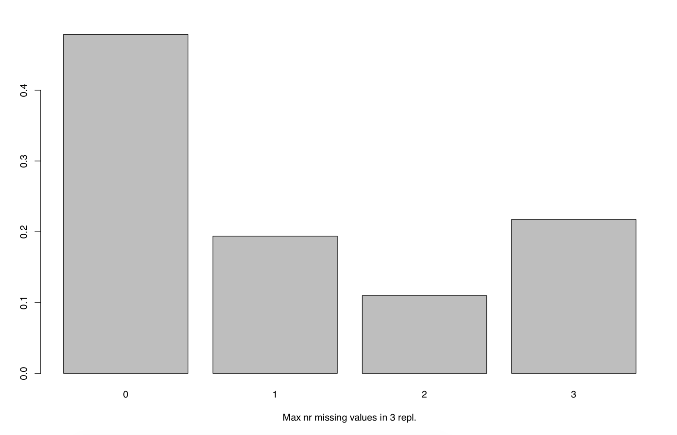 | F 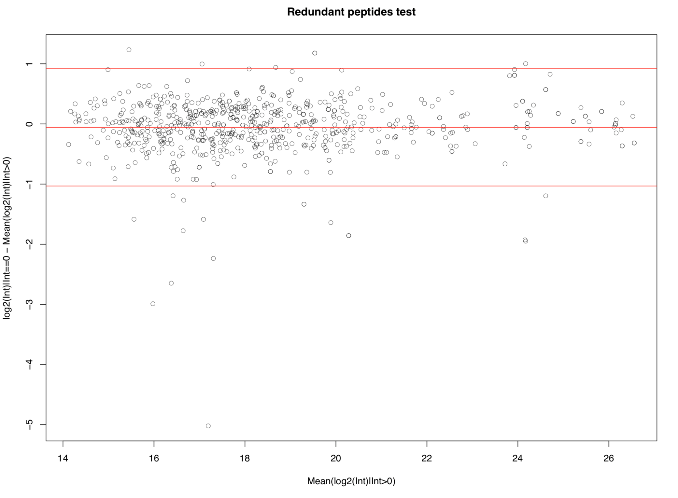 |

**Figure S1: Missing values and their imputation in phospho-proteomics data**A) The occurrence of missing values across the 3000+ phospho-peptides. B) log2-intensity profile of peptide HSS(ph)PDPYGINDKFFDLEK and C) sibling peptide HSS(ph)PDPYGINDK. D) Average log2- intensity of the non-missing values in replicates with 0, 1 and 2 missing values. E) The x-axis shows the maximum number of missing values per 3 replicates in all samples. This barplot displays the frequency of phospho-peptides with a maximum number of 0, 1, 2, and 3 missing values. F) The x-axis is the *α_P’,S,i_* value of all sibling peptides *P*’ without missing values. The y-axis is the difference *d* = *I_P’,S,i_* - *α_P’,S,i_* between the real and estimated value at replicate *i* and sample *S* of *P*’. The horizontal red lines indicate the mean *μ* and *μ±2σ* of the distribution of differences *d*.

**
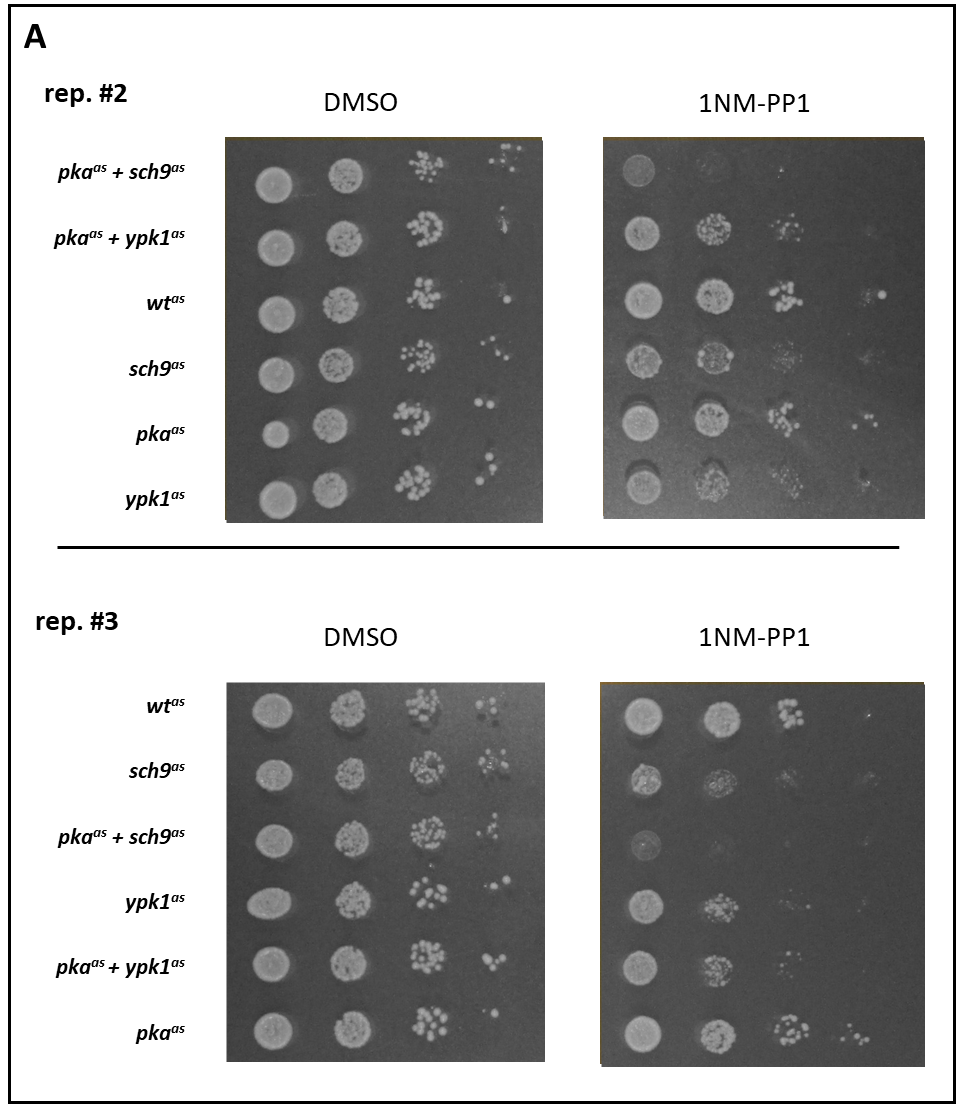

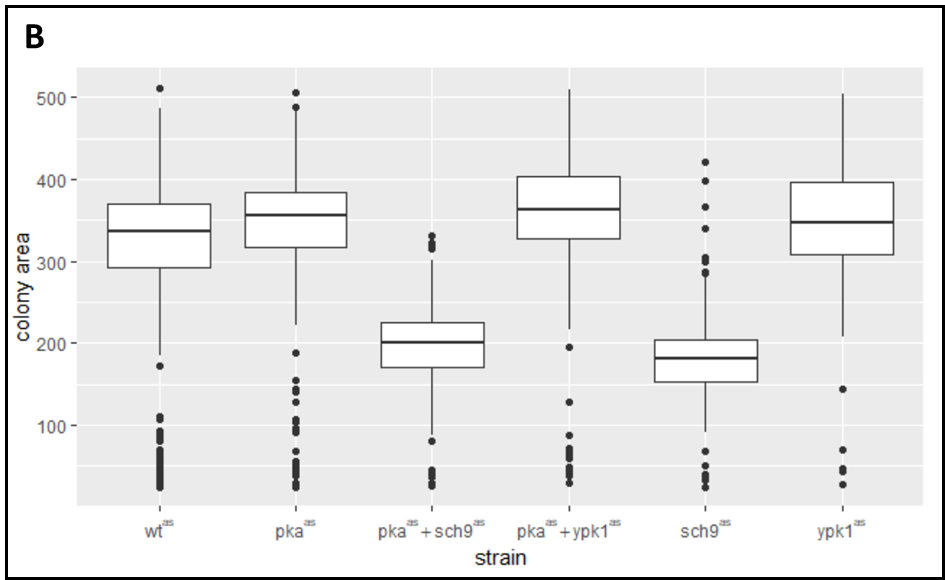
**

**
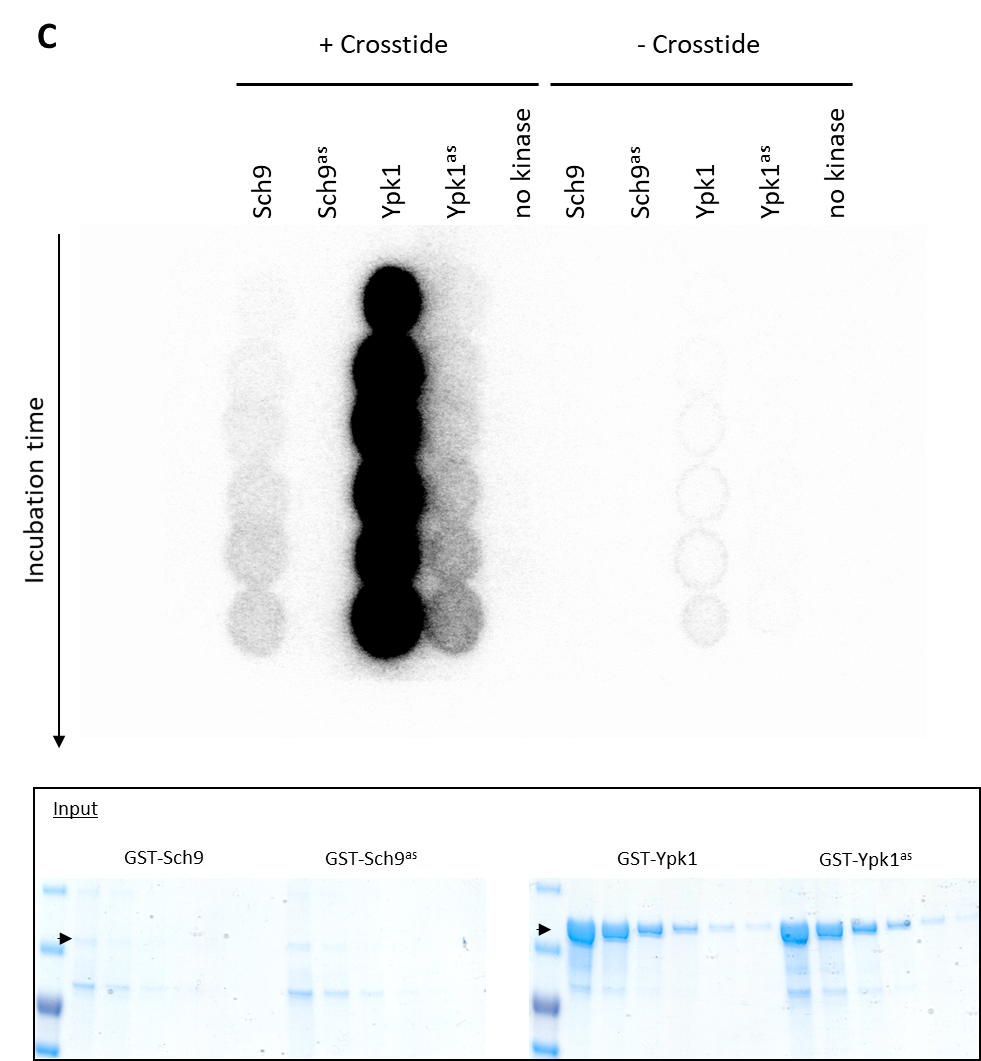
**
**Fig. S2: Effect of analog-sensitive mutations on cell proliferation and kinase activity**A, Additional replicates of experiment depicted in Fig. 1A. Replicate cultures were processed in parallel to samples shown in Fig. 1A and the double analog-sensitive *pka^as^+ypk1^as^* used in Fig. 6 and 7 was tested in addition.

B, Boxplot of colony areas (pixels^2^) of wt^as^ and analog-sensitive strains in absence of 1NM-PP1, determined in an independent experiment.
C, *In vitro* kinase assay using wt or analog-sensitive versions of GST-tagged Ypk1 and Sch9 and Crosstide as a substrate. Samples taken from the kinase assay with increasing incubation time were spotted on filter paper from top to bottom. Lower panel: Coomassie-stained gel of two-fold dilution series of kinase inputs.


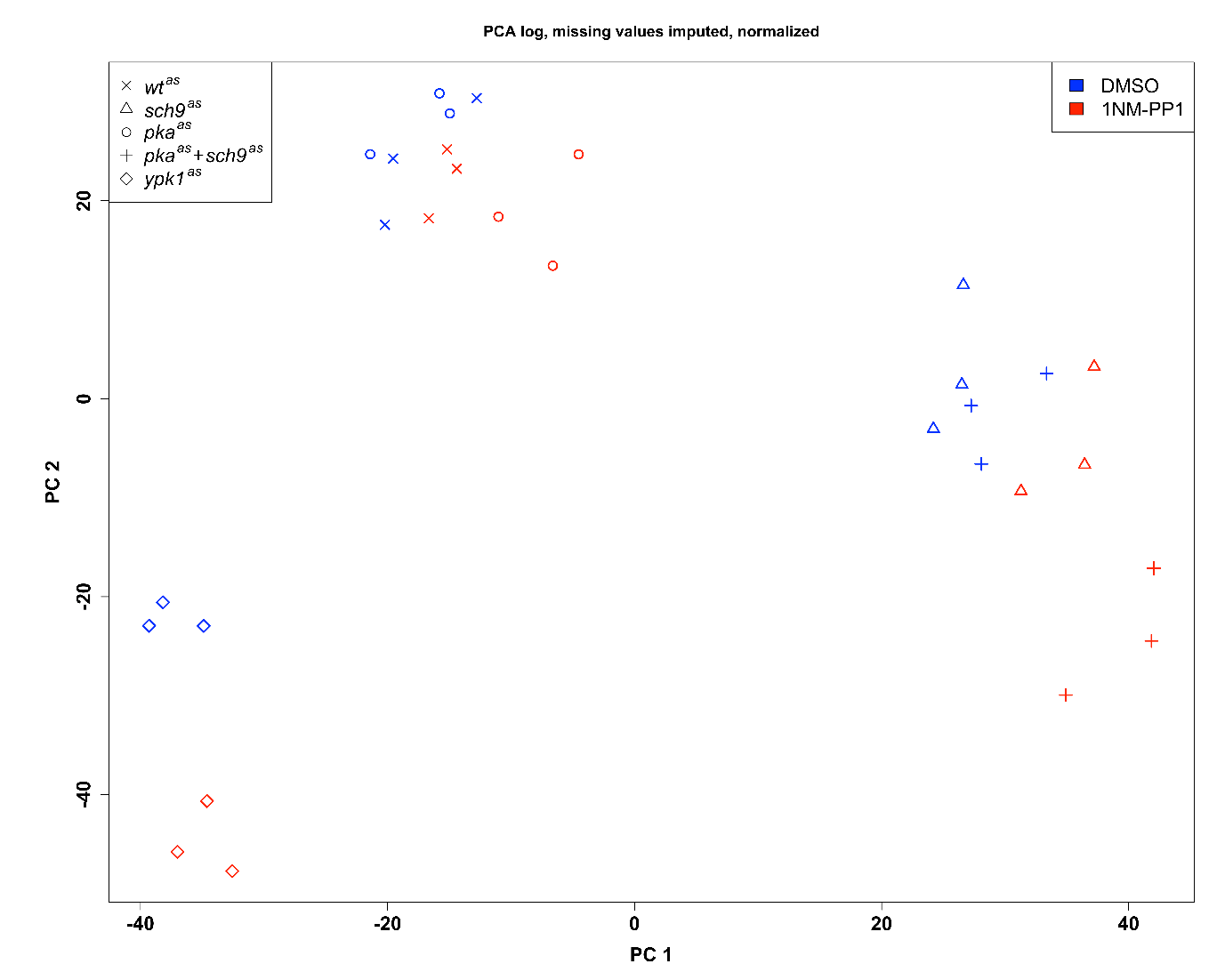


**Fig. S3: Gatekeeper mutations affect the activity of Ypk1 and Sch9**Components 1 and 2 (PC1 and PC2) of principal component analysis of phospho-proteomics data of *wt^as^* and analog-sensitive strains as indicated after 15 min treatment with DMSO (blue) or 500 nM 1NM-PP1 (red).


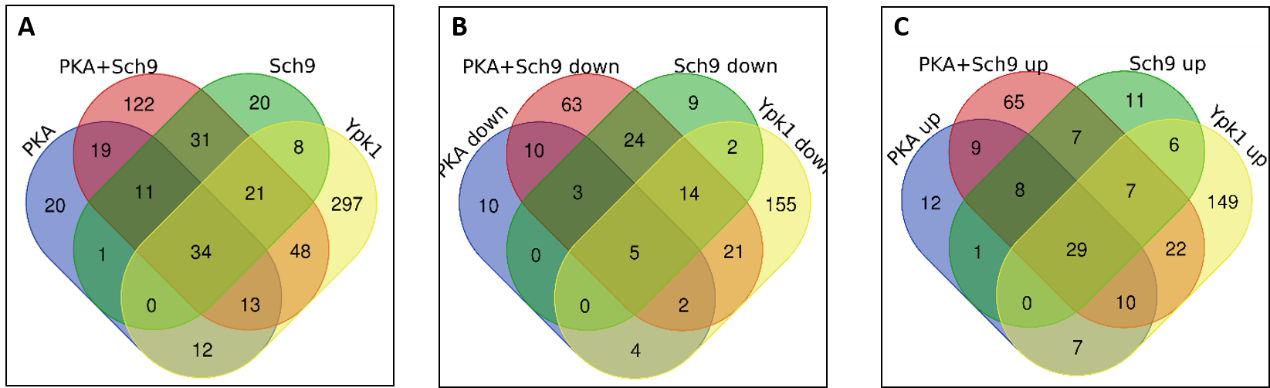

**Fig. S4.** **Overlap of phospho-peptides affected by inhibition of different AGC-kinases.**
Venn diagrams depict phospho-peptides significantly (p_Adj_ < 0.05) affected by inhibition of the kinases indicated. A, Union of hyper- and hypo-phosphorylated phospho-peptides. B, Hypo-phosphorylated phospho-peptides. C, Hyper-phosphorylated phospho-peptides.


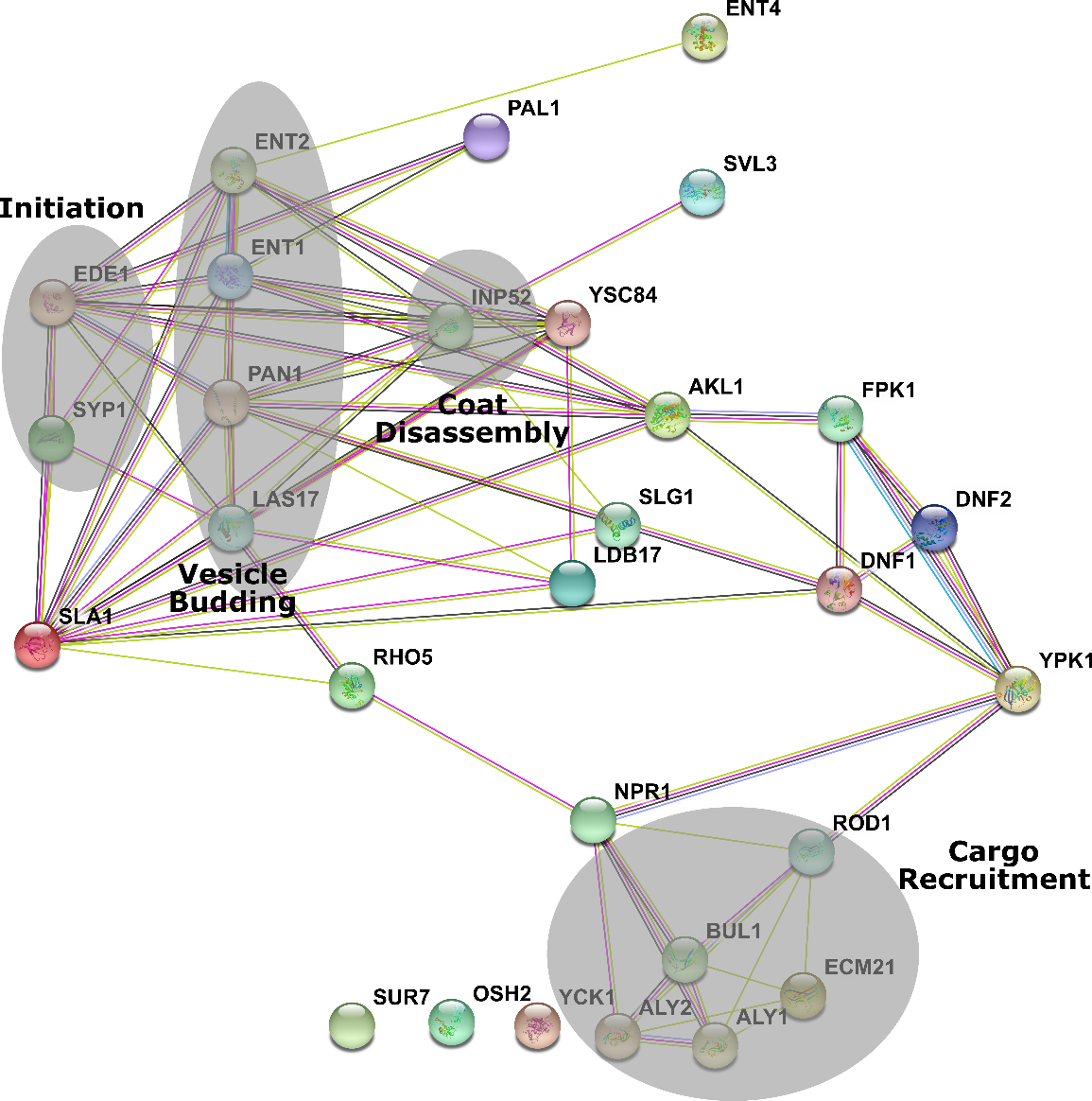


**Fig. S5: Network of endocytotic proteins affected by Ypk1 inhibition.**
Proteins with phospho-sites significantly (p < 0.05) affected by Ypk1 inhibition that are associated with the enriched GO-term “Endocytosis” are displayed as a STRING network map. Proteins associated with initiation of endocytosis, vesicle budding, coat disassembly and cargo recruitment are highlighted.


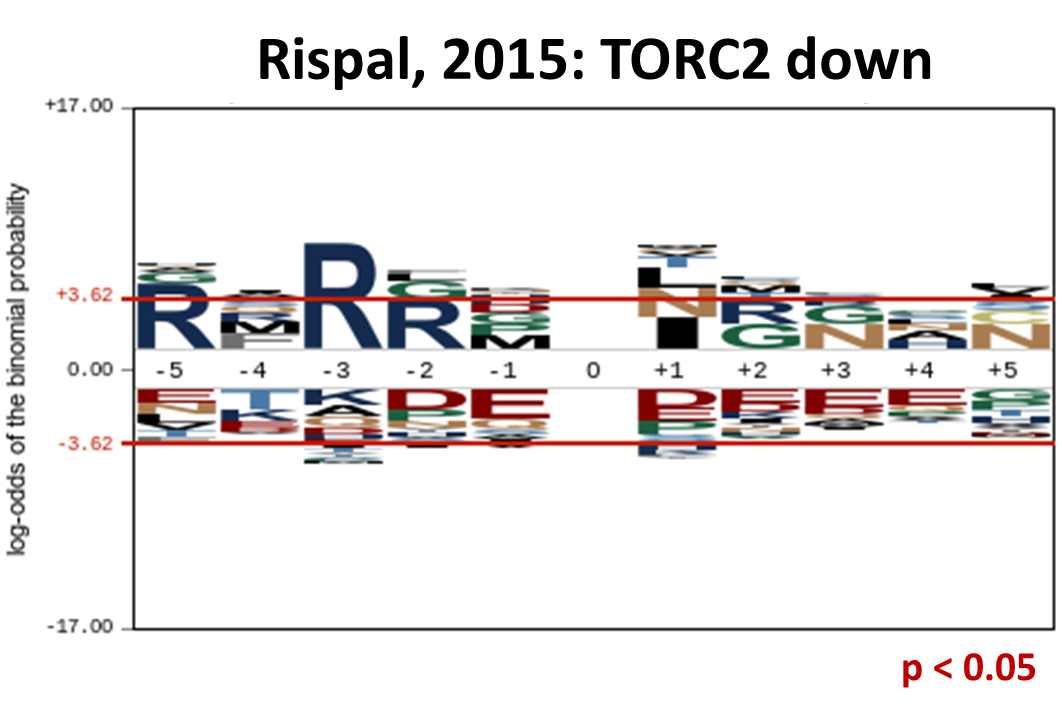
 **Fig. S6: Sequence motif of sites hypo-phosphorylated upon TORC2-inhibition resembles that of Ypk1-inhibition.**A sequence logo was generated from position -5 to +5 around residues found hypo-phosphorylated after 13 min of TORC2-inhibition in Rispal et al., 2015. All phospho-sites quantified in the same study were used as background. Red horizontal lines indicate over- and underrepresentation at p = 0.05.


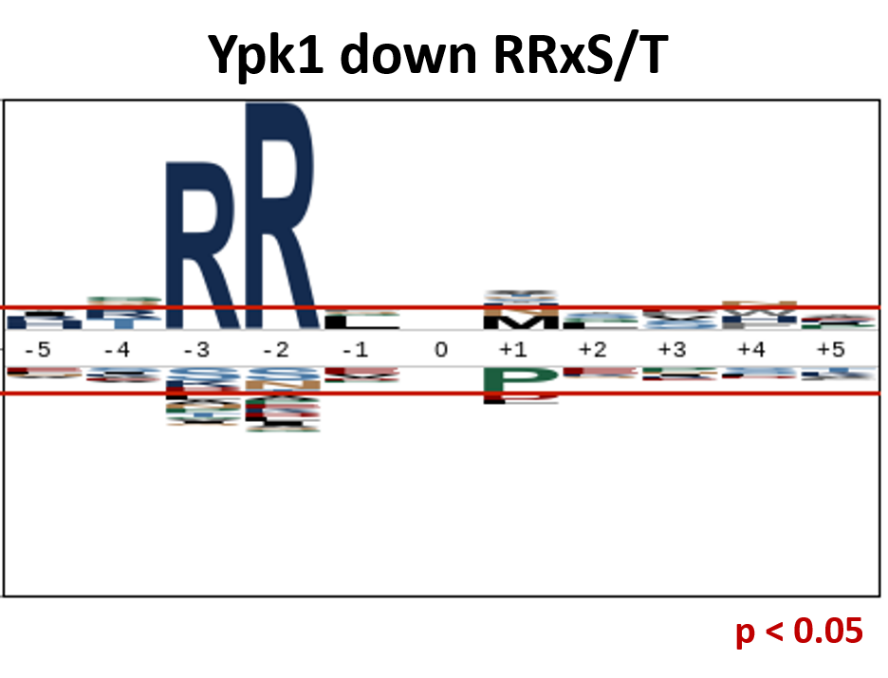

**Fig. S7: No additional amino acids are enriched around RRxS/T phospho-sites hypo-phosphorylated upon Ypk1 inhibition.**A sequence logo was generated from position -5 to +5 around phosphorylated residues using all RRxS/T phospho-sites hypo-phosphorylated upon Ypk1 inhibition as foreground and all quantified phospho-sites as background. Red horizontal lines indicate over- and underrepresentation at p = 0.05.


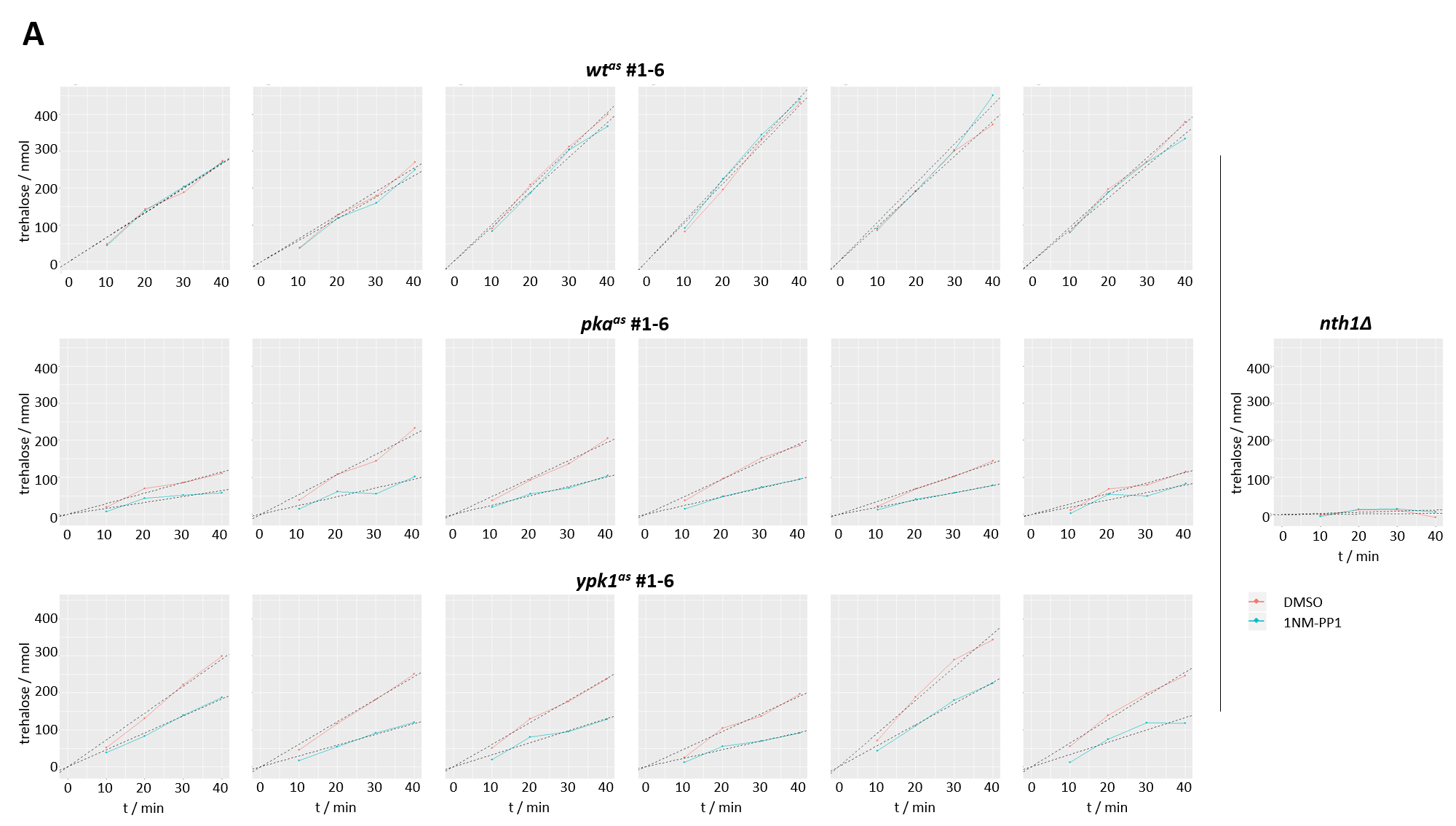


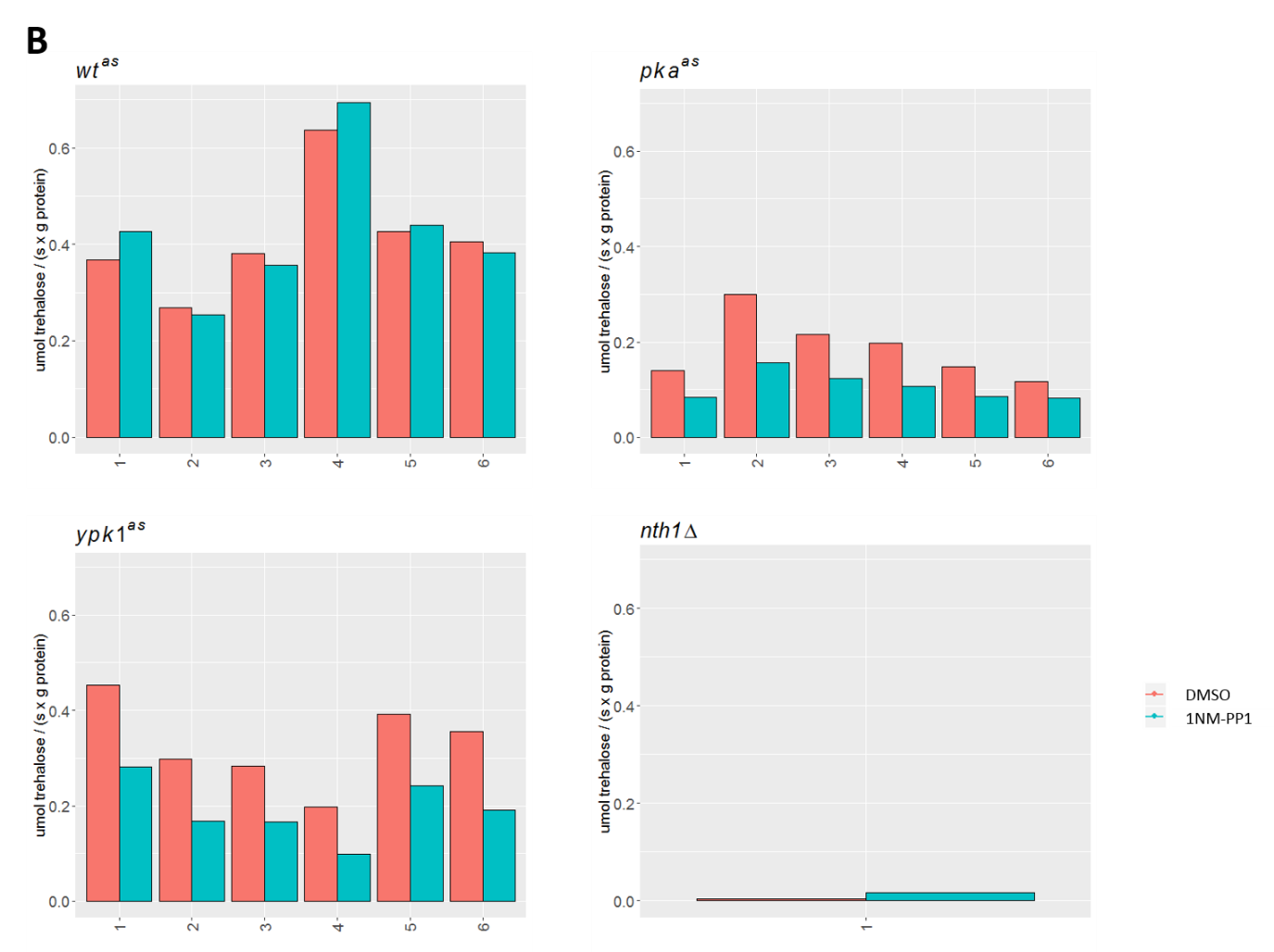


**Figure S8: Inhibition of PKA and Ypk1 leads to reduced Nth1 activity.**Results of trehalase assay depicted in Figure 8.
A, Line graphs depicting amount of trehalose consumed over time after addition of trehalose to permeabilized cell pellets of DMSO (red) or 1NM-PP1 (turquoise) treated *wt^as^*, *pka^as^* or *ypk1^as^* (top to bottom) cultures. Graphs for each of the six replicates for these strains are shown. Dashed lines depict linear fit, the slope of which was used to determine specific trehalase activity. Far right: Corresponding plot for *nth1Δ*-strain.
B, Specific trehalase activity depicting μmol of trehalose consumed per second and per gram of protein is shown as bar plots for the cultures in A.
